# Exploiting gasdermin-mediated pyroptosis for enhanced antimicrobial activity of phage endolysin against *Pseudomonas aeruginosa*

**DOI:** 10.1128/msystems.01106-24

**Published:** 2024-12-23

**Authors:** Dorota Kuc-Ciepluch, Karol Ciepluch, Daria Augustyniak, Grzegorz Guła, Barbara Maciejewska, Artur Kowalik, Ewelina Jop, Zuzanna Drulis-Kawa, Michał Arabski

**Affiliations:** 1 Division of Medical Biology, Jan Kochanowski University in Kielce, Kielce, Poland; 2 Department of Pathogen Biology and Immunology, University of Wroclaw 49572 , Wroclaw, Poland; 3 Department of Molecular Diagnostics, Holy Cross Cancer Centre, Kielce, Poland; Georgia Institute of Technology, Atlanta, Georgia, USA

**Keywords:** *Pseudomonas aeruginosa *LPS, pyroptosis, gasdermin D, endolysin, outer membrane permeabilization

## Abstract

Pyroptosis is an inflammatory immune response of eukaryotic cells to bacterial lipopolysaccharide (LPS) and other pathological stimuli, leading to the activation of the gasdermin D (GSDMD) and secretion of pore-forming domain GSDMD_Nterm_, facilitating the release of cytokines. Additionally, GSDMD_Nterm_ exhibits antibacterial properties through interactions with bacterial outer membranes (OM). We explored alternative antimicrobial strategy to determine whether inducing natural pyroptosis via GSDMD activation by *P. aeruginosa* LPS could enhance the effectiveness of recombinant phage endopeptidase KP27 (peptidoglycan-degrading enzyme) against *P. aeruginosa*, enabling penetration through OM and bacterial killing synergistically. Our findings demonstrated that recombinant GSDMD alone exhibited antibacterial effects against wild-type *P. aeruginosa* with smooth LPS, while recombinant GSDMD_Nterm_ efficiently permeabilized both smooth LPS-bearing and O-chain-deficient *P. aeruginosa* potentially synergizing with endolysin KP27. Transcriptomic analyses revealed the activation of the immune system pathways in response to LPS, mainly in monocytic cells, in contrast to epithelial A549 or HeLa cell lines. LPS-induced pyroptosis in monocytes led to GSDMD cleavage and the release of interleukins, regardless of the nature/origin of the LPS used. However, the pyroptosis stimulation by LPS in the antibacterial assay was not effective enough for bacterial OM permeabilization and enhancement of endolysin activity. We assume that leveraging pyroptosis induction in monocytic cells to augment the bactericidal activity of endolysins may be limited.

**IMPORTANCE:**

Recombinant GSDMD_Nterm_ protein was able to efficiently permeabilize *P. aeruginosa* outer membranes and increase endolysin activity against bacteria, producing either long LPS O-chains or lack them entirely. The obtained results suggest the limited possibility of using the natural process of pyroptosis occurring in monocytic cells to enhance the bactericidal effect of recombinant phage endolysins against Gram-negative bacteria infection.

## INTRODUCTION

Bacterial resistance to antibiotics is a major problem facing society today. Antibiotic-resistant (AMR) bacteria are one of the most serious global threats to humans, which results in the depletion of treatment options, a greater risk of the spread of bacteria, and a longer duration of infection, leading to high morbidity and mortality. AMR bacteria, especially Gram-negative *Pseudomonas aeruginosa*, are responsible for the majority of nosocomial infections, but the number of community-acquired infections that are hard to cure is also increasing (1). The burden of AMR infections forces the search for new alternative treatment solutions. Several alternative antibacterials or virulence-arresting drugs are considered, including nanoparticles, probiotic organisms, bacteriophages, antimicrobial peptides, immunomodulators, and antibodies (2–6). According to the World Health Organization Report on Antibacterial Agents In Clinical and Preclinical Development: An Overview and Analysis (27 May 2022; https://www.who.int/publications/i/item/9789240047655), lytic bacteriophages and phage-derived enzymes have been included in five main groups of non-traditional antibacterials in the pipeline.

One of the most promising is recombinant phage endolysins often used in combination with other drugs or proteins. Endolysins are enzymes hydrolyzing peptidoglycan (PG) and causing bacterial cell lysis. They are necessary for the release of new phage progeny from the infected bacteria (7, 8). Depending on the peptidoglycan cleavage mechanism, we may distinguish five classes of endolysins: lysozymes (N-acetylmuramidases), lytic transglycosylases, glycosidases (N-acetyl-β-D-glucosamidases), N-acetylmuramyl-L-alanine amidases, and L-alanoyl-D-glutamate endopeptidases. Endolysins are a potential antibacterial tool for digesting the peptidoglycan layer and have the advantage of rarely inducing resistance due to their targeting of the conservative bacterial structure (8, 9). Most endolysins originating from Gram-positive-specific phages are modular and composed of enzymatic domain/s (EAD) cleaving PG and a specific cell wall-binding domain/s (CBD) and can be used externally to digest the PG layer (10). Endolysins used by phages targeting Gram-negative bacteria are mostly globular with EAD not effective when applied without (11). The PG of Gram-negative bacteria is hidden beneath the bacterial outer cell membrane (OM), which constitutes a protective barrier limiting the access of endolysins. There are several reports on the effectiveness of endolysins, such as LysSS (12), Lys68, or PlyPAJD-1, against Gram-negative bacteria, including *P. aeruginosa*, especially when combined with cell membrane-disrupting agents (EDTA, citric acid, malic acid, or nigericin), which are able to depolarize and permeabilize the OM (13–15). The disturbance of the integrity of bacterial OM can be also achieved by the application of nanoparticles (16, 17), cationic dendrimers (18, 19), or antimicrobial peptides and proteins (20–24).

An interesting approach is to engage a natural process called pyroptosis, which makes pores in the OM, enabling the phage endolysin to reach the PG. Pyroptosis is an inflammatory type of programmed cell death induced by stimulation of inflammasome formation in eukaryotic cells such as monocytes. Inflammasome complex initiates caspase-1 (canonical) (25, 26) or caspase-4/-5 (non-canonical) (27) signaling and activation of gasdermin D (GSDMD) to form the active N-terminal part of gasdermin D (GSDMD_Nterm_) that triggers the formation of pores in membranes resulting in the leakage of inflammatory cytokines, such as interleukin (IL)-1β and IL-18. The pyroptosis process may be activated by bacterial lipopolysaccharides (LPS), which are one of the most important compounds of bacterial OM, and released outer membrane vesicles interacting with the immune system (28–30). The LPS consists of the core part of the oligosaccharide, the O-antigen, and conserved lipid A, which is responsible for its toxicity (endotoxin) (31). LPSs may activate extracellular receptors Toll-like receptor 4 (TLR4) or intracellular receptors nucleotide-binding oligomerization domain, leucine-rich repeat and pyrin domain-containing protein 3 (NLRP3) inflammasome, which stimulate an inflammation process (32, 33). It has been reported that after bacterial infection and activation of the pyroptotic process, GSDMD can be released outside through the pores and deposited in the OM leading to bacterial lysis (34). This process could be used in conjunction with phage endolysin to enhance the killing effect on bacteria.

The goal of the presented study was to investigate whether *P. aeruginosa* LPS can induce pyroptosis in selected tissue cells and whether it is linked with GSDMD production. If yes, that process could be considered as a natural permeabilization of *P. aeruginosa* OM and might enhance the transport of phage endolysin to periplasmic space for PG degradation, triggering/increasing bacterial lysis during the treatment with endolysin as an antibacterial product.

To verify the aforementioned hypotheses, in the first step of the study, we analyzed the OM permeabilization efficacy of the recombinant GSDMD and GSDMD_Nterm_ on *P. aeruginosa* PAO1 and the ability of gasdermin to increase the recombinant endolysin antibacterial activity. The second part focused on pyroptosis activation and GSDMD expression in different eukaryotic cell lines in the presence of standard commercially available *P. aeruginosa* LPS. This was done at genetic and protein levels to investigate the susceptibility of particular cells to being induced by LPS molecules.

Further, the activation of pyroptosis was performed with previously immuno-characterized LPS of different *P. aeruginosa* strains (PAO1, CF832, CF217, non-CF0038) to check whether there are variations in the proinflammation properties of LPS derived from pathogens retrieved from acute versus chronic infections in CF patients. Finally, we tested the effect of the pyroptosis process induced by selected LPS samples on the antibacterial activity of Klebsiella phage KP27 endolysin.

## MATERIALS AND METHODS

### Bacterial strains and eukaryotic cell lines

*P. aeruginosa* PAO1 (ATCC 15692) wild type and its knock-out Δ*wbpL* mutant deficient in the biosynthesis of A- and B-band O-antigens, provided by Andrew M. Kropinski from the Laboratory of Foodborne Zoonoses, Guelph, ON, Canada, were used. *P. aeruginosa* PAO1, *P. aeruginosa* strains isolated from patients with cystic fibrosis (CF217, CF532-early infection, CF832-late infection, mucoid), and *P. aeruginosa* non-CF0038 strain isolated from burn wound, were from the collection of the Department of Pathogen Biology and Immunology, University of Wroclaw, as described previously (35). LPSs of *P. aeruginosa* O10 and *E. coli* 0111:B4 were purchased from Sigma-Aldrich, St. Louis, USA.

A549 cells (ATCC, Manassas, Virginia, CCL-185), BEAS-2B cells (ATCC, Manassas, Virginia, CRL-9609), HeLa cells (ATCC, Manassas, Virginia, CCL-2), THP1-Xblue cells (Invivogen, Toulouse, France), and THP1-Null2 cells (Invivogen, Toulouse, France) were used in our study. The A549 cells were maintained in F-12K Nutrient Mixture (Kaighn’s modification) with L-glutamine (Corning, New York, USA), HeLa cells in RPMI 1640 with L-glutamine and NaHCO_3_ (Sigma-Aldrich, St. Louis, USA), and THP1-Xblue and THP1-Null2 cells in RPMI 1640 ATTC modified (Gibco, Thermo Fisher Scientific, Waltham, USA).

THP-1 human monocytic cells are the most commonly used model cell line for the study of inflammasome activation. THP1-Null2 cells are derived from THP-1 and produce IL-1β upon stimulation with canonical or non-canonical inflammasome inducers, such as nigericin or LPS. Moreover, they undergo pyroptotic cell death upon inflammasome activation. All cell lines were supplemented with 10% heat-inactivated fetal bovine serum (FBS; Biowest, Nuaille, France) and cultivated at 37°C in a humidified atmosphere of 5% CO_2_.

### LPS isolation and pattern analysis

An examination of the LPS structure pattern was performed using the slightly modified Marolda method of the classical Westphal and Jann protocol, utilizing the hot aqueous phenol extraction (36, 37). The overnight bacterial cultures in the TSB medium (Oxoid, Basingstoke, UK) were centrifuged and adjusted to OD_600_ equal to 2.0 in the phosphate-buffered saline (PBS) buffer. The bacterial cells were disintegrated with lysis buffer (2% SDS, 4% β-mercaptoethanol, Tris, pH 6.8, 1,000°C, 15 min), and the proteins were enzymatically digested with proteinase K (20 mg/mL, 600°C, 1 h). The protein debris was eliminated, and the LPS was isolated by a hot aqueous phenol solution (90%, 70°C, 15 min.). The LPS containing the aqueous phase was purified using ethyl ether to remove residual phenol. To assess the concentration of LPS used for electrophoresis, the purpald method of KDO (3-deoxy-D-manno-oct-2-ulosonic acid) measurement was performed (38). Subsequently, the LPS samples were separated by the Tricine-SDS-PAGE method (14% polyacrylamide gel with 4 M urea, 80 V constant) (37). Finally, after separation, the LPS bands were detected by silver staining (39).

### Cell line propagation for LPS assay

The THP-1 human monocytic cell line (ATCC, TIB-202, Manassas, VA, USA) was cultured in RPMI-1640 medium, (Lonza, BioWhittaker, Verviers, Belgium) supplemented with 10% heat-inactivated human serum (HiFBS, EuroClone, Pero, Italy), 1:100 glutamax (Gibco, Life Technologies, Grand Island, NY, USA), and 1:100 antibiotic–antimycotic solution (Gibco, Life Technologies, Grand Island, NY, USA) at 37°C in the presence of 5% CO_2_. The line was propagated in flasks (SARTSTEDT, Numbrecht, Germany). The number of viable cells was determined by a standard trypan blue exclusion assay using a 0.4% trypan blue solution.

Reporter monocytes of THP1-XBlue cells (InvivoGen) expressing various TLRs and a nuclear factor-kappa B/AP-1-sensitive reporter gene encoding secreted embryonic alkaline phosphatase were cultured at 37°C in a CO_2_ incubator in RPMI 1640 medium supplemented with 10% heat-inactivated fetal bovine serum (30 min at 56°C), 1:100 Glutamax, and 200 µg/mL of zeocin. The cells were passaged every 3 days by inoculating 7 × 10^5^ cells/mL.

### Cytokine stimulation and detection for LPS characteristics

The method was performed according to our protocol described previously (40). A total of 5 × 10^5^ THP1 cells in 0.5 mL of medium were incubated with various concentrations of isolated LPS, in duplicate for 24 h in 24-well microplates (NUNC, Thermo Fisher Scientific, Roskilde, Denmark) at 37°C with 5% CO_2_. After incubation, cells were centrifuged at 1,200 × *g* for 5 min, and culture supernatants were harvested, centrifuged, pooled, aliquoted, and stored frozen (−80°C). Enzyme-linked immunosorbent assays (ELISAs) for human IL-8 and tumor necrosis factor alpha (TNF-α), were performed using serially diluted samples according to the manufacturer’s instructions (DuoSet ELISA, R&D, Minneapolis, USA).

### Hemolytic assay for LPS characteristics

Sheep blood stabilized in Alsever’s solution was purchased from Proanimali (Wroclaw, Poland) and stored at 4°C before use. Of blood sample, 7 mL was centrifuged at 1,500 × *g* for 10 min, and the resulting plasma fraction, together with leukocytes, was discarded from the samples. The pellet was washed with PBS (pH 7.4), mixed by inversion, and centrifuged. After five washing steps, the pellet of sheep red blood cells (SRBC) treated as 100% was diluted to a concentration of 1.6% (vol/vol) in veronal buffer (pH 7.4) referring to 8 × 10^8^ cells/mL. Of 1.6% SRBC, 150 µL was mixed in a 1:1 ratio with various concentrations of LPS in 2-mL Eppendorf tubes and incubated for up to 2 h in a water bath at 37°C. Then, SRBC were centrifuged (1,500 × *g*, 5 min, room temperature [RT]), and 200 µL of hemolytic supernatant was transferred to a 96-well flat-bottom microplate. One percent Triton X100 and veronal buffer were used as positive and negative (diluent) controls, respectively. The percentage of hemolysis was calculated from a 10-point hemolytic standard curve constructed as follows: 0.8% SRBC cell lysate obtained by mixing 2.4% SRBC and 2 mL of miliQ referring to 100% hemolysis diluted serially to obtain standards of hemolysis in the range 10%–100%. Each standard in duplicate was applied to the 96-well flat-bottom microplate, and the absorbance was measured at 541 nm on a Varioskan Lux multimode multi-plate reader (Thermo Fisher Scientific, Waltham, USA).

### Residual hemolytic activity of complement for LPS characteristics

1. Sensitization of SRBC by hemolysin (rabbit IgG anti-SRBC): In a Falcon tube, 2.4% SRBC (vol/vol) in veronal buffer were mixed with 2,500× diluted hemolysin in a ratio of 1:1 and incubated for 15 min at RT;
2. Activation of serum complement by LPS: LPS at 1 mg/mL was preincubated with normal human serum (NHS) in a 1:9 ratio for 30 min at 37°C in a water bath;
3. The residual activity assay: The residual activity of LPS-pretreated NHS was determined by complement hemolytic assay (CH50). LPS-pretreated NHS was initially diluted 20× with veronal buffer and then used in the following reaction mixtures in Eppendorf tubes: (A) 100 µL of NHS, (B) 80 µL of NHS +20 µL of veronal buffer, (C) 60 µL of NHS +40 µL of veronal buffer, (D) 60 µL of NHS +40 µL of veronal buffer. To each tube, 200 µL of IgG-sensitized SRBC was added, and the whole was incubated for 30 min at 37°C in a water bath. Then SRBCs were centrifuged (1,500 × *g*, 5 min, RT) and the extent of cell lysis was determined by transferring 200 µL of hemolytic supernatant to a 96-well flat-bottom microplate, and absorbance was measured at 541 nm on a Varioskan Lux multimode multi-plate reader (Thermo Fisher Scientific, Waltham, USA). Each assay contained a negative control (spontaneous hemolysis control of sensitized SRBC with buffer) and a positive control (hemolysis of sensitized SRBC in the presence of non-treated NHS). The percentage of hemolysis from tubes A, B, C, and D, was calculated from a 10-point hemolytic standard curve. The complement activity was expressed as activity units (AU) referring to the reciprocal of the serum dilution that yields 50% hemolysis of SRBC.

### Complement serum bactericidal assay

Active normal sheep serum (NSS) as a source of complement was purchased from the provider of animal biospecimens ProAnimali (Wroclaw, Poland) and stored at −80°C. To determine sensitivity to serum, bacteria from overnight liquid culture at TSB were centrifuged, washed with saline, and diluted to ∼5 × 10^5^ CFU/mL of saline. The 100 µL of bacterial suspension was mixed at a 1:1 vol/vol ratio with active NSS or heat-inactivated NSS (56°C, 30 min) serum to obtain 50% serum concentration and incubated for 2 h at 37°C in a water bath. At times 0, 1, and 2 h, bacteria were serially diluted and plated (10 µL) on Tryptic Soy Agar plates for CFU/mL calculation.

### Recombinant endolysin preparation

The recombinant phage-borne endolysin was prepared according to the method described previously by Maciejewska et al. (41). Briefly, the coding sequence of Klebsiella phage KP27 endopeptidase (endolysin) was amplified using Pfu polymerase (ThermoFisher Scientific, Waltham, MA, United States) and cloned into the commercially available pEXP-5-CT/TOPOR TA expression vector (Invitrogen, Thermo Fisher Scientific, Waltham, USA) according to the manufacturer recommendations. The expression was conducted for 18 h at 20°C using *E. coli* BL21 (DE3) pLysS (Agilent Technologies, Santa Clara, CA, United States) and isopropyl-b-D-1-thiogalactopyranoside (final concentration of 0.5 mmol/L) as an inductor of the expression. The recombinant protein was purified from the filtered supernatant by affinity chromatography using NGC medium pressure chromatography systems (Bio-Rad, Hercules, CA, United States) combined with 5-mL nickel columns using Bio-Scale Mini Profinity IMAC Cartridges (Bio-Rad, Hercules, CA, United States) and dialyzed against a PBS buffer. The concentration of purified recombinant enzyme was then determined fluorometrically (QubitR Protein Assay Kit, Molecular Probes, Thermo Fisher Scientific, Waltham, MA, United States).

### Permeabilization of the bacterial OM by GSDMD

Permeabilization of *P. aeruginosa* strains PAO1 and its knock-out Δ*wbpL* mutant was tested after adding commercially available GSDMD protein (Origene, Rockville, USA; NM_024736) or GSDMD_Nterm_ obtained after incubation with 0.1 U of caspase 4, and without/with 36 µg/mL of Klebsiella phage KP27 endopeptidase. The experiment was performed using the N-phenyl-1-naphthylamine (NPN) probe uptake assay. The bacteria were grown at 37°C to obtain the appropriate optical density (OD_600_ = 0.5), and the bacterial pellet after centrifugation was dissolved in HEPES buffer (correspondingly 1 mL of the buffer per 0.5 mL of bacteria before centrifugation) to obtain OD_600_ = 0.1. The measurement was performed in a sterile 96-well plate. The NPN fluorescent dye was added to the bacterial suspension in the well (10 µM per well). Then, appropriate concentrations of GSDMD and endolysin were added to the mixture. NPN fluorescence intensity was monitored for 30 min with a time interval of 1 min at an excitation wavelength of 350 nm and an emission wavelength of 420 nm using a TECAN Infinite 200 PRO microplate reader (Tecan Group Ltd., Switzerland). The results were presented in relative fluorescence units (RFU).

### Quantitative reverse transcription PCR (RT-qPCR)

RT-qPCR was used to test the effect of *P. aeruginosa* O10 LPS on *GSDMD* gene expression in THP1-Xblue cells. The cells were incubated with LPS at a concentration range of 0.001–100 µg/mL for 24 h in a humidified atmosphere of 5% CO_2_. Total RNA was extracted from THP1-Xblue and THP1-Null2 using SV Total RNA Isolation System (Promega, Madison, USA), RNeasy Plus Mini Kit (Qiagen, Hilden, Germany), or using TRI REAGENT (Sigma-Aldrich, St. Louis, USA) form three independent repeats. The amount of RNA was assessed quantitatively and qualitatively using Nanodrop Spectrophotometer (Thermo Scientific, Waltham, USA), where the RNA quality for all samples was between 1.8 and 2.0, and by RNA integrity number (RIN), where the value of RIN was between 8 and 10 for all samples using Bioanalyzer (Agilent, St. Clara, USA). Reverse transcription was performed using the Reverse Transcription System (Promega, Madison, USA) following the manufacturer’s protocol with random hexamer primers. One microliter of cDNA solution of each sample was used for qPCR in a 20-µL reaction mixture containing the following: 10 µL of 2× SsoAdvanced Universal SYBR Green Supermix (Bio-Rad, Hercules, USA), 1 µL of PrimePCR SYBR Green Assay: GSDMD (Bio-Rad, Hercules, USA), 1 µl of PrimePCR SYBR Green Assay: ACTB (β-actin) (Bio-Rad, Hercules, USA), and 8 µL of dH_2_O. The relative expression level of β-actin was used as a control. The following conditions were used: 95°C for 2 min. by one cycle, 95°C for 5 s, and 60°C for 30 s by 40 cycles using the equipment Eco Real-Time PCR (Illumina, San Diego, USA). Experiments were done in three independent repeats. *ACTB* gene was used as an internal reference gene, and the ΔΔCT method was used for relative quantification.

### DNA microarray assay

Total RNA was extracted from A549 and HeLa cells using the isolation kits Sv Total system RNA Isolation (Promega, Madison, USA) and Rneasy Plus Mini Kit (Qiagen, Hilden, Germany), and from THP1-Xblue cells using TRI REAGENT (Sigma-Aldrich, St. Louis, USA) from three independent replicates according to the manufacturer’s protocol. The quality and quantity of RNA were checked using the Nanodrop Spectrophotometer (Thermo Scientific, Waltham, USA) and by the RNA integrity number (Agilent, St. Clara, USA) as described in Quantitative reverse transcription PCR (RT-qPCR). Two-Color Microarray-Based Gene Expression Analysis assay (Agilent, St. Clara, USA) was used for the analysis of gene expression in A549, HeLa, and THP1-Xblue cells treated with 0.1 µg/mL of *P. aeruginosa* O10 LPS in comparison to non-treated control, according to the manufacturer’s instruction. The expression of RNA was measured using DNA chips of Agilent numbers US11233913_257236328396_S02_GE2_1010_Sep10, US11233913_257236328397_S02_GE2_1010_Sep10, and US11233913_257236328398_S01_GE2_1010_Sep10, which contained probes for approximately 56,000 human genes along with long non-coding RNA (lncRNA). The experiment was three times replicated as biological replicates. The analysis of Microarray data was made using the special gene expression software GeneSpring GX suitable for DNA Microarray instruments from Agilent.

Total RNA was amplified and labeled using Low Input QuickAmp Labeling Kit Two-Color (Agilent Technologies, Santa Clara, CA, US), following the manufacturer’s instructions. The labeled cDNA was synthesized from 50 mg of total RNA using 5× first-strand buffer, 0.1 M DTT, 10 mM dNTP mix, and Affinity Script RNase Block Mix according to the manufacturer’s procedures. The samples were incubated at 40°C for 2 h, and the reverse transcription and dsDNA synthesis were terminated by incubating at 70°C for 15 min. The dsDNA transcription was performed by adding Transcription master mix (5× Transcription buffer, 0.1 M DTT, NTP mix, T7-RNA polymerase, and Cyanine 3/5-CTP thereby incorporating Cy3 [control samples] or Cy5 [test samples]-labeled dCTPs) to the dsDNA reaction samples and incubating at 40°C for 2 h. Amplified and labeled cRNA was purified on an RNase mini column (Qiagen) according to the manufacturer’s protocol. After checking the labeling efficiency, the cyanine-3- and cyanine-5-labeled cRNA were mixed, and cRNA fragmentation was performed by adding 10× Gene Expression Blocking Agent and 25× Fragmentation Buffer and incubating at 60°C for 30 min. Fragmented cRNA was resuspended in 2× Hi-RPM Hybridization Buffer and directly pipetted onto the SurePrint G3 Human Array Microarray (Agilent Technologies). The array was hybridized at 65°C for 17 h using a hybridization oven (Agilent Technology, USA). The hybridized microarrays were washed as per the manufacturer’s washing protocol (Agilent Technologies). The hybridization images were analyzed by a DNA microarray scanner (Agilent Technologies).

The analysis of microarray data was performed using GeneSpring GX software (Agilent, St. Clara, USA). The statistical analyses of the microarray assay were calculated by the gene expression software GeneSpring GX (Agilent, St. Clara, USA). Data were interpreted by comparison with a control group using a *t*-test with Benjamin–Hochberg false discovery rate (FDR) correction, and the significance level was set at *P* < 0.05. Gene ontology was analyzed using the Quick GO, and pathway analysis was performed based on the Reactome databases with Benjamin–Hochberg FDR correction and *P* < 0.05. The Panther databased was used for establishing significantly changed molecular function and biological processes. Additionally, genes showing statistically significant changes (*P* < 0.05) obtained by comparing the expression between LPS-exposed and non-exposed cell lines were analyzed using the PathfindR R package. The PathfindR package uses an active subnetwork-oriented pathway enrichment analysis to place a set of genes of interest in the pathway context (https://pubmed.ncbi.nlm.nih.gov/31608109/).

### ELISA for pyroptosis detection

THP1-Xblue cells were seeded at a density of 10^6^ cells per well. The cells were incubated with *P. aeruginosa* O10 LPS in the concentration range of 0.001–1 µg/mL. The medium was collected after 24 h. The supernatants were used for the ELISA test according to the manufacturer protocol (Human IL-1 beta ELISA Kit; Abcam, Cambridge, Great Britain). The concentration of IL-1β in the supernatants was calculated according to standard curves. The results of the amount of IL-1β are shown in μg/mL of supernatant. Experiments were done in three independent repeats.

### Western blot

In the first step, THP1-Null2 cells were pre-incubated with 1, 10, or 100 µg/mL of *E. coli* 0111:B4 LPS for 3 h; incubated with nigericin at 10 µM for 24 h; and supernatants were collected to check the possibility of GSDMD induction. In the second part of the study, THP1-Null2 cells were pre-incubated with 1 µg/mL of *P. aeruginosa* O10, PAO1, CF532, CF832, CF217, and non-CF0038 LPS; incubated with nigericin at 10 µM for 24 h; and the supernatants were collected. Western blot technique was performed according to Bio-Rad (Hercules, USA) company protocol, using their chemicals. Approximately 20 µL of supernatant with 20 µL of Laemmil protein sample buffer were loaded on Mini-PROTEAN TGX Gel 4%–20% SDS-PAGE for electrophoresis followed by electro-transfer to nitrocellulose. The membranes were blocked in 3% bovine serum albumin in TBST (TRIS-buffered saline with Tween 20 buffer) and then incubated overnight with the following primary antibodies: anti-GSDMD (GSDMDC1, sc-81868, Santa Cruz Biotechnology, Dallas, USA) and anti-glyceraldehyde-3-phosphate dehydrogenase (anti-GAPDH; 1:1,500 dilution; Abcam, Cambridge, Great Britain; ab8245 and ab9485) at 4°C. After washing with TBST, the membranes were incubated in HRP (horseradish peroxidase)-conjugated secondary antibody solution (1:3,000 dilution; Abcam, Cambridge, Great Britain; ab6721) and RBTXMS IGG (H + L) HR (1:200 dilution; Life Technologies, Carlsbad, USA; 31450) for 1 h at room temperature. Signals were detected with chemiluminescence. GAPDH was used as a control protein to normalize the target protein. As a positive control, 0.75 µM of GSDMD recombinant protein (Origene, Rockville, USA; NM_024736) was used.

### Antimicrobial activity assay

The THP1-Null2 cell line was treated with a final concentration of 1 µg/mL of LPS of *P. aeruginosa* (PAO1, CF532, CF832, CF217, non-CF0038) and of *E. coli* 0111:B4 and/or 10 µM nigericin (pyroptosis positive control). Then, the THP1-Null2 cell line supernatants, containing GSDMD oligomers, were used for antimicrobial activity assay. It was tested alone or in combination with KP27 endolysin against the *P. aeruginosa* PAO1 strain. In detail, a bacterial culture of 10^4^ CFU/mL in TSB medium was incubated for 18 h at 37°C in the presence of 2× diluted cell line supernatant alone or with 36 µg/mL of KP27 endolysin. The effect was measured using OD_600_. The experiments were done in three biological repeats. One-way analysis of variance (ANOVA) with *post-hoc* Dunnett’s test was used for statistics.

### Statistical analysis

STATISTICA version 13.3 (StatSoft, Poland) was used for statistical data analyses of RT-qPCR, ELISA, and antibacterial studies. The normal distribution of variables was verified using the Shapiro–Wilk test, whereas the homogenicity of variance was tested by the Brown–Forsyth test. Data from RT-qPCR and antibacterial studies were established using ANOVA with *post-hoc* Dunnett’s test. Results from ELISA were analyzed using ANOVA with *post-hoc* Tukey test. The results are expressed as the mean ± standard deviation (SD). The significance level was considered for *P* < 0.05 (*).

## RESULTS

**Fig 1:**
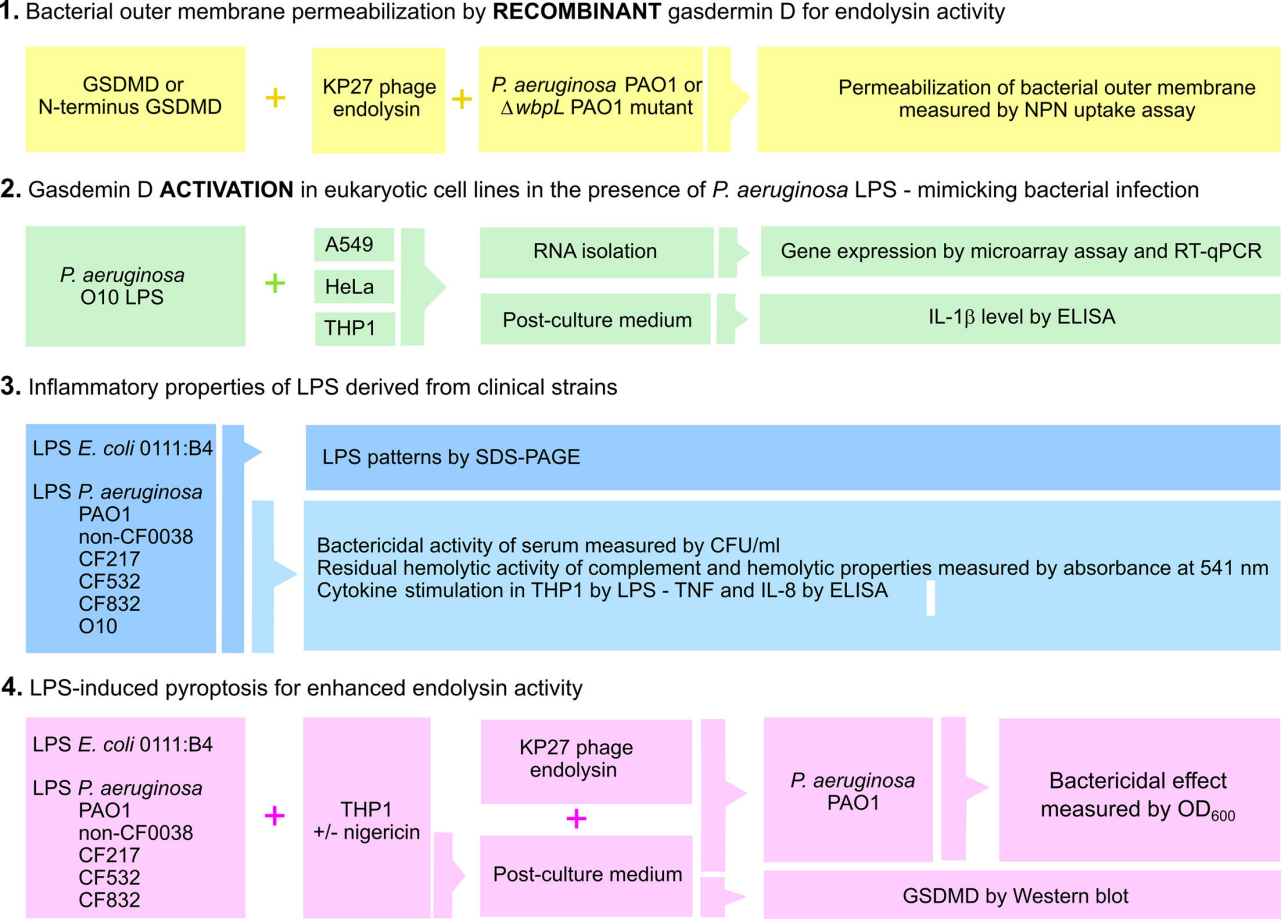
The experimental pipeline of the study (1). Recombinant gasdermin D (GSDMD) or its N-terminal region for membrane permeability of *P. aeruginosa* PAO1 and its LPS O-chain-deficient mutant (*ΔwbpL*) was monitored using NPN uptake assay. Membrane permeability experiments were performed for GSDMDs alone and in the presence of KP27 endolysin (2). Mimicking bacterial infection—gasdermin D activation by *P. aeruginosa* O10 LPS in A549, HeLa, or THP1 cells was measured by microarray (gene expression) and ELISA (IL-1β) assays (3). The inflammatory properties of LPS isolated from *E.coli* (control) and tested *P. aeruginosa* strains were evaluated using immune-serological tests. The patterns of isolated LPS samples (the O-chain presence or absence) were characterized using the SDS-PAGE method (4). LPS-induced pyroptosis alone or in combination with nigericin was tested on the THP1 cell line to obtain active gasdermin D for enhanced KP27 endolysin activity against *P. aeruginosa* PAO1 strain. LPS was isolated from tested *P. aeruginosa* strains and *E. coli* (control). Gasdermin (GSDMD) production was monitored using Western blot, and the antibacterial activity of endolysin was evaluated using culture optical density (OD_600_).

The study was designed in four steps to investigate the natural pyroptosis process as an anti-pseudomonal immune response potentially supporting the antibacterial activity of PG-degrading endolysin derived from phages (Fig. 1).

### Gasdermin is able to permeabilize *P. aeruginosa* OM, but only GSDMD_Nterm_ increases endolysin activity

**Fig 2:**
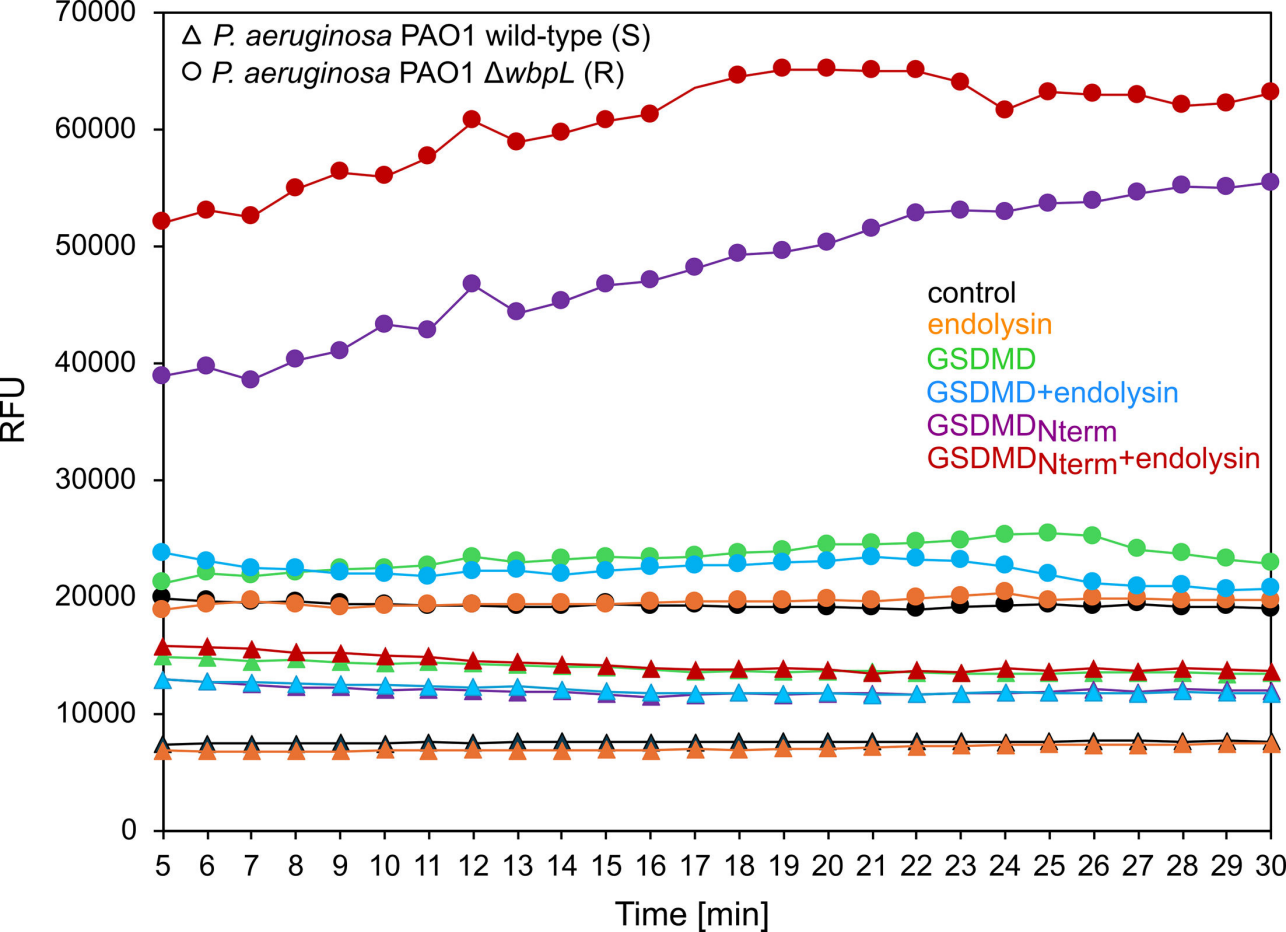
OM permeabilization of *P. aeruginosa* PAO1 wild type with LPS (**S**) and its *ΔwbpL* mutant lacking LPS O-chain (**R**) by 1.5 µM GSDMD or GSDMD_Nterm_ recombinant proteins alone or in combination with endolysin (36 µg/mL) calculated as the relative fluorescence units (RFU). The control consisted of untreated *P. aeruginosa* cells.

To study whether GSDMD affects phage endolysin activity against *P. aeruginosa*, the fluorescent NPN probe uptake method was used. This assay is based on the assumption that intact OM is a permeability barrier blocking the influx of hydrophobic substances such as NPN. Once the OM is damaged, the NPN enters the phospholipid layer resulting in prominent fluorescence (42). This step verified the membrane-permeabilizing activity of commercially available recombinant GSDMD and GSDMD_Nterm_ obtained after cleavage by caspase-4, while combined or not with phage KP27 endolysin. PAO1 and its *ΔwbpL* mutant were used to check whether the O-chain presence or absence affects GSDMD interaction with bacterial membranes and the formation of pores. Our results showed that both GSDMD and GSDMD_Nterm_ used alone were able to permeabilize *P. aeruginosa* PAO1 wild-type cells with smooth LPS (Fig. 2). This effect was potentiated for GSDMD_Nterm_ and weakened for GSDMD in the presence of KP27 endolysin. For the *ΔwbpL* mutant cells lacking the O-chain, only GSDMD_Nterm_ was able to permeabilize bacterial membrane (Fig. 2), and this effect was enhanced in combination with KP27 endolysin. It suggested that GSDMD and GSDMD_Nterm_ might have different mechanisms for membrane permeabilization. GSDMD can increase the membrane’s permeability *via* electrostatic/hydrophilic interactions promoted by LPS O-chain, which is disturbed in the presence of endolysin. Moreover, the lack of membrane permeabilization in the *ΔwbpL* mutant (R form) confirmed the role of O antigen in GSDMD activity. In contrast, GSDMD_Nterm_ could permeabilize the membrane of both S and R bacteria and this effect was potentiated in the presence of endolysin.

### *P. aeruginosa* LPS induces various responses in different eukaryotic cell lines with the strongest upregulation in monocytic cells

After the confirmation that extracellularly supplied GSDMD and GSDMD_Nterm_ can form pores in *P. aeruginosa* OM, we next verified whether GSDMD activation is possible in a natural pyroptosis process *via P. aeruginosa* LPS. For this purpose, the following three cell types that potentially could be exposed to *P. aeruginosa* in the infection process were chosen: the well-defined monocytic cell line (THP1-Xblue), epithelial human lung cells A549, and epithelial human uterus/cervix cells HeLa (43–46). All cell lines were treated with 0.1 µg/mL of standardized commercial LPS of *P. aeruginosa* O10 serotype, and the gene expression profile was studied by microarray assay as well as RT-qPCR technique.

**Fig 3:**
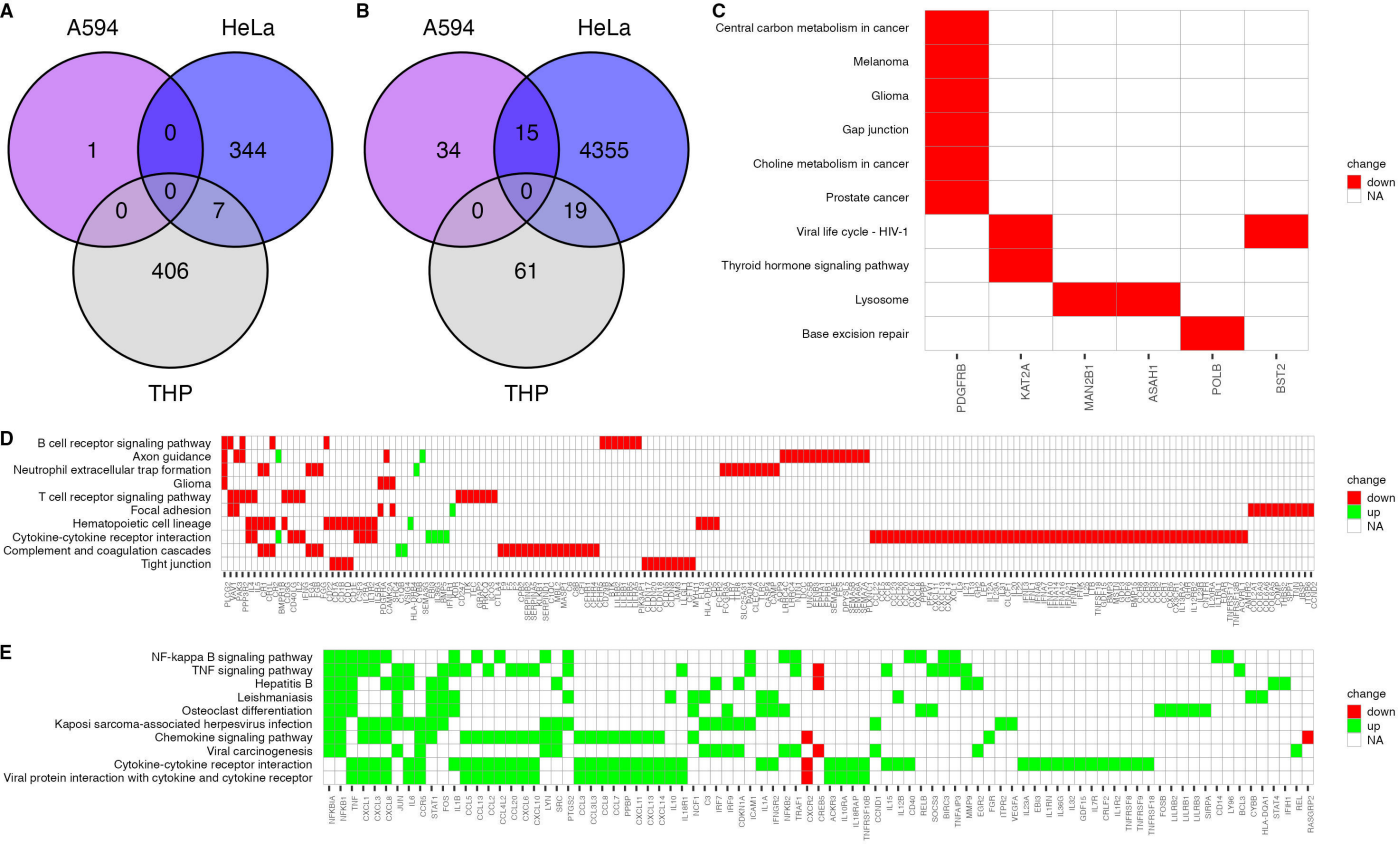
Two-color microarray-based gene expression analysis of cell lines treated with *P. aeruginosa* O10 LPS. The number of up- (**A**) and downregulated (**B**) genes in tested cell lines. Heatmaps of KEGG pathway enrichment analysis (PahthfindeR, R package), based on comparison of expression between LPS-exposed and non-exposed cell lines, are presented for A549 (**C**), HeLa (**D**), and THP (**E**).

Using a Two-Color Microarray-Based Gene Expression Analysis, 50 differentially expressed genes (DEGs) were identified with significant fold changes (*P* ≤ 0.05) in A549 cells (Fig. 3A and B), and the pathway enrichment analysis (PahthfindeR, R package) based on comparing expression between LPS-exposed and non-exposed A549 cells line is presented in Fig. 3C. The analysis using the Reactome software showed only the *SSBP3-AS1* gene with an upregulated expression, which encodes a putative uncharacterized protein with an unknown function. The gene ontology (GO) terms of downregulated genes were associated with the G protein-coupled receptor signaling pathway, positive regulation of calcium ion import, and positive regulation of MAP kinase activity (kappaB kinase/NF-kappaB signaling positive regulation of ERK1 and ERK2 cascade chemokine-mediated signaling pathway, cellular response to interferon-gamma, immune response, and protein binding (Table S1, Supporting Data). Generally, the response of A549 to LPS was low because epithelial cells do not express the CD14 receptor engaged in the LPS signal transduction pathway (47, 48). In contrast, the enrichment of cancer-associated pathways (melanoma, glioma, prostate cancer, central carbon metabolism in cancer, and choline metabolism in cancer) and viral infection (viral life cycle- HIV-1) was observed in the A549 cell line (Fig. 3C).

For HeLa cells, 4,740 genes (351 upregulated, 4,389 downregulated) were identified with significant fold changes (*P* ≤ 0.05) (Fig. 3A and B). The analysis of GO terms for HeLa cells using the Reactome software revealed upregulated expression of the G protein-coupled receptor signaling pathway, G protein-coupled receptor activity, DNA binding, regulation of transcription, ion transport and binding, and negative regulation of the apoptotic process. Only two genes were related to GO terms such as innate immune response, antibacterial humoral response or immunoglobulin production involved in immunoglobulin-mediated immune response (Table S2, Supporting Data). The GO terms of downregulated genes were associated with ion binding and transport, negative regulation of inflammatory response, response to transforming growth factor beta, and protein binding (Table S3, Supporting Data). The level of gene downregulation was 10 times higher than its upregulation in HeLa cells evoked by LPS. The analysis using PahthfindeR (R package) of the pathway enrichment shows that mainly immune system response mechanisms are downregulated in the presence of *P. aeruginosa* O10 LPS (Fig. 3D). It seems possible that LPS decreases the production of cellular components involved in the immunity of HeLa cells. Moreover, enrichment of pathways related to the immune system (B-cell receptor signaling pathway, neutrophil extracellular trap formation, T-cell receptor signaling pathway, cytokine–cytokine receptor interaction, complement, and coagulation cascades) and intercellular junctions (focal adhesion, tight junctions) were blocked in the HeLa line.

In the case of THP1-Xblue cells, 493 genes (413 upregulated, 80 downregulated) were identified with significant fold changes (*P* ≤ 0.05) (Fig. 3A and B). The top 15 upregulated genes in THP1-Xblue with the highest fold change are listed in Table S4 (Supporting Data), which include *CXCL13*, *CXCL3*, *CCL2*, *IL1β*, *RGS1*, *MIR146A*, *TNFAIP6*, *CCL8*, *CXCL8*, *MPP9*, *CCL7*, *IL6*, *CXCL1*, *SOCS3*, and *CCL20.* These genes are generally associated with a cellular and inflammatory response to LPS, including IL-1β specific to pyroptosis. The top 15 downregulated genes in THP1-Xblue with the highest fold change are listed in Table S5 (Supporting Data), which include *MS4A3*, *SERPINB10*, *GPA33*, *CLEC12A*, *CNR1*, *DEPDC4*, *SLC40A1*, *TMOD1*, *DSC3*, *NMUR1*, *RET*, *CLRN1*, *F13A1*, *SERPINI2*, and *MS4A6A.* These genes encode integral components of membrane-associated proteins generally involved with signal transduction, signaling, disease, metabolism of protein, metabolism of RNA, hemostasis, immune system, and metabolism. Moreover, gene lists obtained by comparing gene expression of THP1-Xblue cells exposed to LPS compared with that of the same cell line without this exposure were analyzed using the Pathfinder R package (Fig. 3E). For the THP1-Xblue cell line, enrichment of pathways related to the immune system activity (NF-kappa B signaling pathway, TNF signaling pathway, chemokine signaling pathway, and cytokine–cytokine receptor interaction) and viral or protozoan infection (hepatitis B, leishmaniasis, Kaposi sarcoma-associated herpes virus infection, viral carcinogenesis, and viral protein interaction with cytokine and cytokine receptor) was observed.

Taking into account both analyses by the Reactome software and Pathfinder R package, the activation of pathways associated with the action of the immune system in response to LPS can be observed mainly in monocytic cells. The heatmaps with the general gene expression profile, including all upregulated and downregulated genes in THP1-Xblue cells generated from DNA microarray data and GeneSpring GX software, are presented in Fig. S1 (Supporting Data).

**Fig 4:**
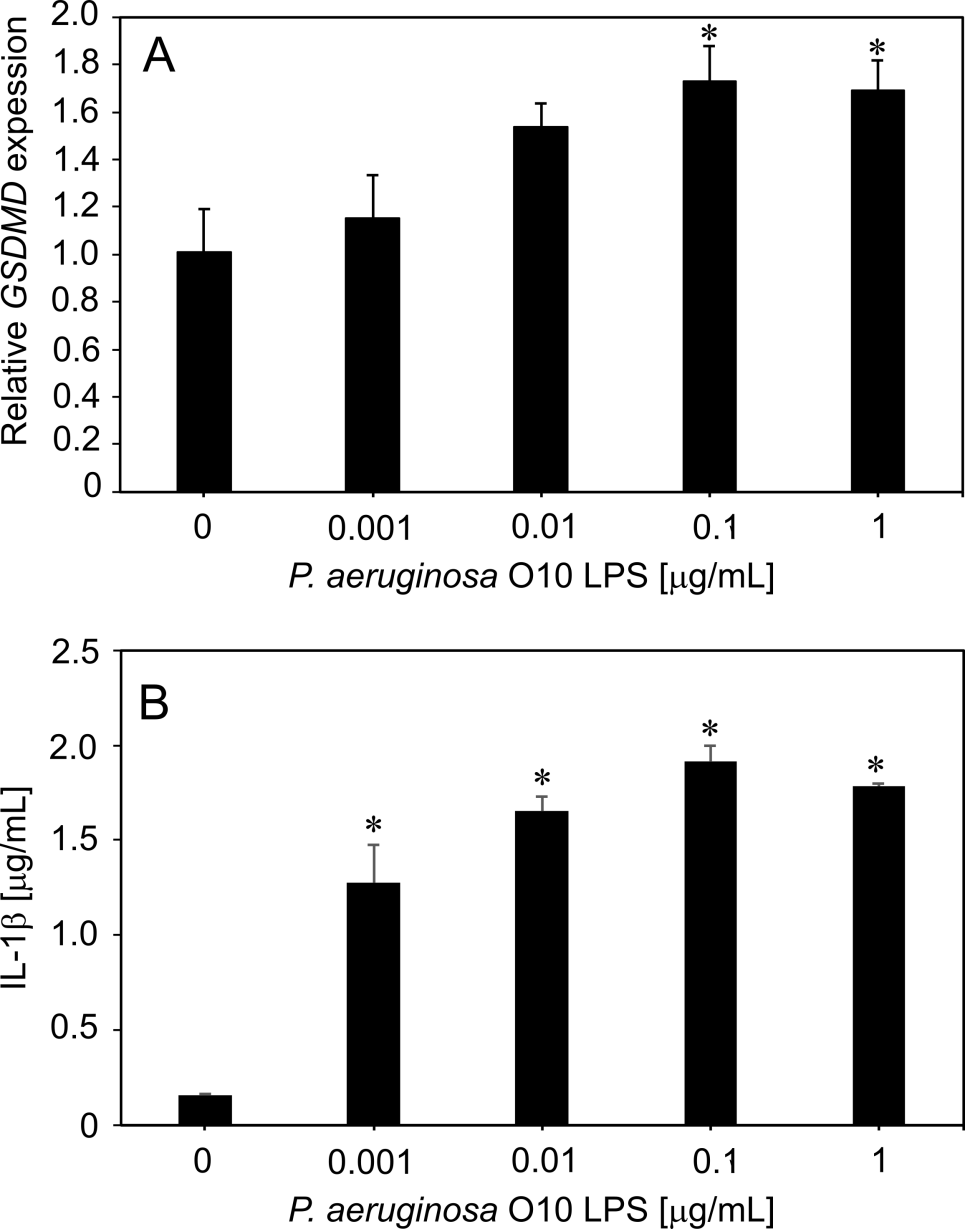
The induction of pyroptosis in monocytic cells by *P. aeruginosa* O10 LPS. The GSDMD expression level analysis by RT-qPCR in THP1-Xblue cells after treatment with serial concentrations of O10 LPS. The ACTB gene was used as an internal reference gene, and the ΔΔCT method was applied for relative quantification (*P < 0.05) (**A**). The IL-1β production by THP1-Xblue cells in the presence of *P. aeruginosa* O10 LPS measured using ELISA; *P < 0.05 (**B**). One-way ANOVA with Dunnett’s *post-hoc* test was used to test the statistically significant differences.

The DNA microarray analyses showed that the tested LPS induced the strongest upregulated immune response in THP1 cells, including inflammatory response to LPS (IL-1β) specific to pyroptosis, compared to epithelial A549 or HeLa cell lines. Nevertheless, the gene expression profile from the DNA microarray assay did not show statistically significant *GSDMD* gene overexpression relative to the control. We hypothesized that there is a specific mechanism for limiting GSDMD transcript expression or GDSMD degradation to protect the cell against self-lysis caused by multiple pore formation regardless of LPS concentration. To double check that phenomenon, the RT-qPCR method was utilized for the *GSDMD* gene expression in THP1-Xblue cells treated with serial concentrations of *P. aeruginosa* O10 LPS (Fig. 4A). The RT-qPCR confirmed the increased *GSDMD* gene expression level in monocytic cells but only for ≥0.1-µg/mL concentrations of *P. aeruginosa* O10 LPS. It might indicate that a detectable level of GSDMD transcript is an effect of limited mRNA expression or its degradation to protect cells from self-lysis *via* pyroptosis.

Simultaneously as a pyroptosis marker, the level of IL-1β was determined by ELISA test in the post-culture medium of THP1-Xblue cells treated with LPS. It turned out that the induction of IL-1β production in THP1-Xblue cells was seen for ≥0.001 µg/mL of *P. aeruginosa* O10 LPS (Fig. 4B). It seems important that LPS at lower concentrations was able to induce IL-1β production in contrast to GSDMD mRNA expression in THP1-Xblue cells. This effect might also be explained by the limitations of ELISA assay, which, in fact, may detect biologically inactive precursors (pro-IL-1β) (49).

### Inflammatory properties of LPS derived from clinical strains

In this step, we checked whether LPS derived from various *P. aeruginosa* strains, including those from clinical sources (acute infections and cystic fibrosis), could differ in the inflammatory properties and thus potentially also in the ability to induce GSDMD synthesis by monocytic cells *via* induction of pyroptosis. Several LPS features impacting immunoreactivity were tested for selected strains (PAO1, non-CF0038, CF217, CF532, and CF832). Their LPS differed in terms of the following: (i) the presence of long O chain and most strains had smooth type (S), while CF217 strain lacked O antigen—rough type (R) (Fig. S2), (ii) hemolytic properties with LPS from CF217 as the strongest inducer of hemolysis (Fig. S3), and (iii) sequestration of complement components with LPS from strain CF217 as the weakest complement consumer (Table S6). The full activity of serum complement against CF217 was confirmed in a 50% serum-dependent bactericidal assay showing its complement-sensitive phenotype (Table S7).

**Fig 5:**
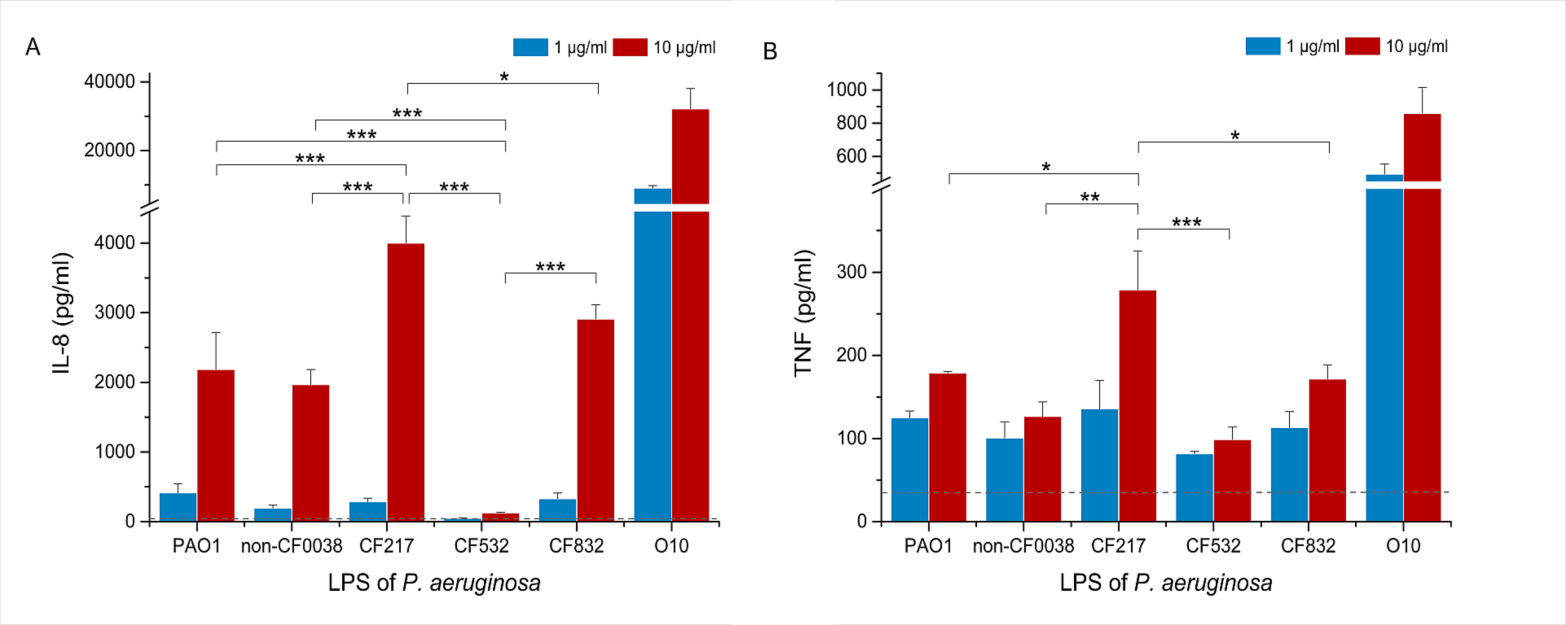
Induction of inflammatory response of THP-1 monocytes by *P. aeruginosa* LPS. Supernates from 24-h THP-1 cultures stimulated with various concentrations of LPS were evaluated using ELISA for the presence of proinflammatory cytokines (**A**) IL-8 and (**B**) TNF. Data are expressed as mean ± SEM from at least two independent experiments performed in two plates at four technical repeats each (two technical repeats per plate). Supernates from duplicates from each plate were pooled and assessed using ELISA. Dashed lines refer to non-stimulated control. One-way ANOVA with *post-hoc* Tukey test was used to test the differences. *P < 0.05, **P < 0.01, ***P < 0.0005. As a positive control, LPS from O10 strains was used.

Likewise, the common proinflammatory properties of the LPS tested, including the induction of TNF and IL-8 release by THP-1, were dependent on the LPS nature/origin (Fig. 5).

A comparison of LPS-dependent IL-8 release showed significant differences depending on the source of LPS used, but only at the higher (10 µg/mL) concentration of endotoxin (Fig. 5A). In this case, the strongest stimulator was LPS from the strain CF217, and the weakest was LPS from the non-mucoid CF532 strain. Analogous one-way ANOVA analyses of TNF also showed significant differences for higher concentrations of LPS used; however, the results indicated that, of the clinical isolates, only the rough LPS CF217 had the strongest pro-inflammatory properties, whereas proinflammatory potential of other LPS was at a similar level (Fig. 5B). At lower 1-µg/mL concentrations of LPS, no differences were observed between the LPS tested in the induction of pro-inflammatory cytokines. Compared to the unstimulated control, the presence of TNF in the culture supernatant was sevenfold higher, while IL-8 was 100-fold higher.

The highest inflammatory response was shown for commercial control O10 LPS. Summing up, LPS from various clinical strains in higher concentrations have different immunoreactive and proinflammatory properties as reflected by IL-8 release.

### LPS-induced pyroptosis for enhanced endolysin activity

**Fig 6:**
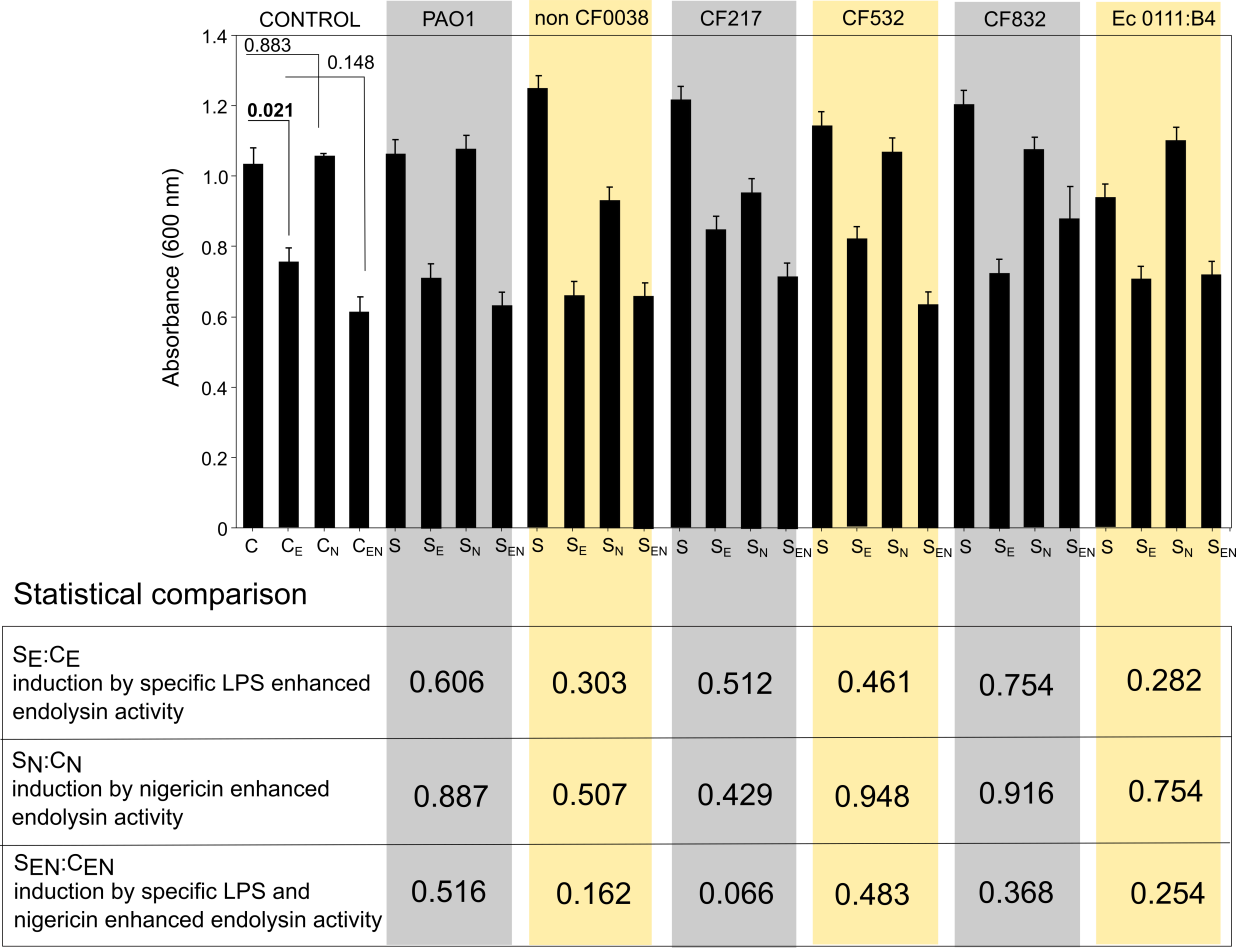
The OD_600_ of *P. aeruginosa* PAO1 culture growth was measured in TSB supplemented with 2× diluted THP1-Null2 cell line supernatant, either alone or with 36 µg/mL of KP27 endolysin (**E**) after 18 h of incubation at 37°C. The THP1-Null2 line was first pre-treated with 1 µg/mL of LPS (**S**) and/or 10 µM nigericin (**N**). C, bacterial culture treated with THP1-Null2 supernatant; C_E_, bacterial culture treated with THP1-Null2 supernatant and endolysin; C_N_, bacterial culture treated with THP1-Null2 supernatant after nigericin stimulation; C_EN_, bacterial culture treated with THP1-Null2 supernatant after nigericin stimulation and supplemented with endolysin; S, bacterial culture treated with THP1-Null2 supernatant after LPS stimulation; S_E_, bacterial culture treated with THP1-Null2 supernatant after LPS stimulation and supplemented with endolysin; S_N_, bacterial culture treated with THP1-Null2 supernatant after LPS and nigericin stimulation; S_EN_, bacterial culture treated with THP1-Null2 supernatant after LPS and nigericin stimulation and supplemented with endolysin. Color columns show particular LPS samples used for pyroptosis induction. The experiments were done in three biological repeats, and statistical data were established by one-way ANOVA with *post-hoc* Dunnett’s test. The *P*-values are presented in the bottom table.

To study the potential variation in propyroptotic activity of the aforementioned LPS and influence of LPS-induced GSDMD release on enhanced endolysin activity, we investigated the antibacterial effect of recombinant Klebsiella phage KP27 endopeptidase against *P. aeruginosa* PAO1 in the presence of post-LPS-induced THP1 culture media. *P. aeruginosa* PAO1 culture reduction was measured by the optical density at 600 nm (Fig. 6). KP27 endolysin in post-culture THP1 medium without LPS stimulation showed moderate but significant antibacterial effect against PAO1 bacterial cells (*P* = 0.021). It turned out that THP1-Null2 post-culture media after stimulation by tested LPS samples, alone or in combination with nigericin, did not significantly increase the antibacterial effect of endolysin in comparison to the relevant controls indicating at least an insufficient level of pore-forming GSDMD forms.

To verify the presence of pore-forming GSDMD in tested supernatants, the Western blot technique was applied to detect the GSDMD protein and its cleavage products in a post-culture medium after incubation of THP1-Null2 with the LPS samples (Fig. S4). The GSDMD was detected in the control growth medium, which could suggest that GSDMD might be produced constitutively by THP1-Null2 cells as an inactive pro-protein crucial for the biochemical "ready to react" system in response to bacterial infection (LPS) *via* pyroptosis. Moreover, the full GSDMD protein was also observed after treatment with *E. coli* 0111:B4 LPS at 1–100 μg/mL (Fig. S4A) and different *P. aeruginosa* LPS at 1 µg/mL (Fig. S4B). Theoretically, GSDMD (484 amino acids residues; 53 kDa) in response to LPS should be cleaved by inflammatory caspases (caspase-1 and -4/-5/-11) to the GSDMD C-terminus domain (GSDMD_Cterm_, 276–484 amino acids residues; 22 kDa) and the GSDMD N-terminus domain (GSDMD_Nterm,_ 1–275 amino acids residues; 31 kDa) forming pores in the membrane to enable the release of inflammatory cytokines IL-1β and IL-18 (50, 51).

By testing only the supernatants in our assay, we were able to detect only the soluble fractions of GSDMD and GSDMD_Cterm_, with the latter one as the indirect proof for the formation of membrane interacting GSDMD_Nterm_ likely associated with the lipid fraction of the cells not tested here.

Interestingly, in all samples and controls, GSDMD (53 kDa) and GSDMD_Cterm_ (22 kDa) were detected. An additional band (~30 kDa) was noted after administration of *E. coli* 0111:B4 LPS alone at a higher concentration (100 µg/mL), and a lower concentration (10 µg/mL) post nigericin stimulation (Fig. S4A). Moreover, ⁓40 kDa was also seen for *E. coli* 0111:B4 LPS at 100 µg/mL in combination with nigericin. Those bands might represent the GSDMD_Cterm_ aggregates formed in response to increased stimulation of THP1 cells by concentrated LPS combined with nigericin.

In general, the results suggest that GSDMD cleavage is observed in THP1-Null2 overexpressing the inflammasome system and that 1 µg/mL of LPS derived from *E. coli* or different *P. aeruginosa* strains did not stimulate monocytes to increase the formation of the pore-forming GSDMD domain.

## DISCUSSION

Bacteria and their components (LPS) are capable of activating the pyroptosis process (an inflammatory form of cell death) in the eukaryotic immune response (52). In this process, the gasdermin D (GSDMD) protein is activated and cleaved to N- and C-terminal regions followed by GSDMD_Nterm_ accumulation and pore formation in the membrane required for the release of cytokines such as interleukin IL-1β and IL-18 (52–54). Simultaneously, a formed GSDMD_Nterm_ serves as an antibacterial response thanks to the interactions with bacterial OM, pore formation, and bacterial lysis (51, 55). Assuming that process, the pyroptosis leading to bacterial OM permeabilization may help the externally applied endolysin to reach the periplasmic space and peptidoglycan; thus, the combination of gasdermin (GSDMD) and endolysin could act synergistically in phage-based therapy.

In this work, we studied whether the induction of the natural pyroptosis process through the activation of GSDMD could, first, be achieved by *P. aeruginosa* LPS, and, second, enhance the anti-pseudomonal effect of recombinant phage endopeptidase KP27.

Initially, commercially available GSDMD and GSDMD_Nterm_ were tested together with endolysin KP27 on two *P. aeruginosa* strains PAO1 and Δ*wbpL* to determine the possibility for enhancing enzyme activity (8, 56). Our study showed that GSDMD alone has an antibacterial effect only on the wild-type PAO1 possessing the smooth LPS (S), but not on the O-chain-deficient mutant. The antibacterial activity of GSDMD might be associated with its protein structure containing two different antibacterial peptides. Kuand and coworkers showed that 80–90 amino acid residues (peptide 1) and mainly 70–90 amino acids of peptide 2, with a flexible loop present in the GSDMD, can inhibit the growth of *E. coli* and *Mycobacterium smegmatis* (50). In our study, GSDMD could permeabilize *P. aeruginosa* cells by the interaction of those peptide regions with bacterial surface structures. Interestingly, GSDMD-mediated bacterial cell membrane permeabilization in the *P. aeruginosa* PAO1 wild type was reduced in the presence of KP27 endolysin, probably by the enzyme interactions either with the LPS O-chain masking the available moieties for GSDMD peptides or directly with the GSDMD molecules.

In contrast, we proved that the GSDMD_Nterm_ domain was able to permeabilize both *P. aeruginosa* with smooth LPS as well as O-chain deleted mutant. GSDMD_Nterm_, as a full protein (GSDMD)-cleaving product by caspase-1 (1–275 amino acids residues), can form pores in the membrane in contrast to full GSDMD protein (51). The presence of these additional regions in GSDMD_Nterm_ close to peptides 1 and 2 might promote higher interaction with a membrane (phosphatidylserine and cardiolipin), which could support the activity of endolysin in effective membrane permeabilization. Indeed, such an effect was observed for both smooth WT PAO1 and Δ*wbpL* mutant lacking an LPS O-chain.

The first line of humoral innate immune defense against Gram-negative bacteria regards usually the serum complement activity; therefore, most *P. aeruginosa* produces long O-chains to escape permeabilization by the membrane attacking complex (MAC) of an activated complement. The results obtained in the above experiments suggest hypothetically that the alternative or additional defense mechanism effective against bacteria with all types of LPS (long or short) is based on gasdermin D activity induced during pyroptosis.

Another aspect elucidated in our study concerned the verification of whether GSDMD activation takes place in a pyroptosis process induced by *P. aeruginosa* LPS. Three cell types stimulated by 0.1 µg/mL of *P. aeruginosa* O10 LPS were used for transcriptomic analyses, including epithelial human lung cells (A549), epithelial human uterus/cervix cells (HeLa), and monocytic cells (THP1), to mimic bacterial infection. The same concentration of *E. coli* LPS was significant in the studies of Eder et al. (57).

Transcriptomic analyses revealed that the activation of pathways associated with the action of the immune system in response to LPS can be observed mainly in monocytic cells in contrast to epithelial A549 or HeLa cell lines. It seems reasonable that epithelial tissues are not intensively proinflammatory stimulated by the presence of Gram-negative bacteria while having constant contact with the natural microbiome.

The analyses showed that upregulated genes of THP1 were associated with several significant GO terms associated with cellular response to lipopolysaccharides (26, 28). Nevertheless, no statistically significant *GSDMD* gene overexpression relative to the control was detected in monocytic cells, which we hypothesized to be related to the protection against cell self-lysis caused by multiple pore formation. Indeed, the RT-qPCR confirmed the increased *GSDMD* gene expression level in monocytic cells but only for ≥0.1-µg/mL concentrations of *P. aeruginosa* O10 LPS used in microarray assay. It might indicate an effect of limited mRNA expression or its degradation to protect cells from self-lysis *via* pyroptosis.

In the next step, we checked whether the immune response, including pyroptosis (31, 45), can be achieved in the human monocytic cells after induction by different LPS derived from *P. aeruginosa* strains (O10, PAO1, non-CF0038, CF217, CF532, CF832) and *E. coli* LPS (0111: B4). Additionally, other immunoreactivity of tested LPSs were also examined to elucidate variations in proinflammatory properties. A comparison of LPS-dependent IL-8 release showed significant differences depending on the source of LPS used, but only at a higher concentration of endotoxin indicating that the strongest stimulator was LPS from strain CF217, and the weakest was LPS from the non-mucoid CF532 strain. In the case of TNF, only the rough LPS CF217 had the strongest pro-inflammatory properties, whereas the proinflammatory potential of other LPS was on a similar level. Interestingly, *P. aeruginosa* undergoes lipid A structural modification during CF chronic infection, and this altered lipid A structure induces stronger signaling through TLR4, which is reflected in the production of proinflammatory cytokines (58). Excluding the rough LPS from CF217, the other smooth LPSs showed only subtle differences in complement sequestration that were not correlated with the sensitivity of particular strains in complement-mediated bactericidal tests. The reason for that could be not only the different lengths and density of O-antigen but also O-antigen composition and chemistry within the studied LPS (59, 60). It is logical, therefore, that a similar activation of the complement does not necessarily result in its lytic effect if, for example, the overall LPS structure block MAC (C5b-9) insertion or surface mucoid properties shields the outer membrane from lytic complement activity (non-mucoid CF532 versus mucoid CF832).

The above-described well-defined LPS samples were then used to stimulate pyroptosis in THP1 cells to enhance the antibacterial activity of phage endolysin KP27. It turned out that although the endolysin alone exhibited moderate activity against PAO1 strain, no synergistic effect was noted for the combination with supernatants of LPS-induced monocytic cells, which indicates at least an insufficient level of pyroptosis event and bacterial OM permeabilization by GSDMD.

The Western blot experiments confirmed that GSDMD protein is synthesized on a constant level in THP1 cells, and its soluble domain GSDMD_Cterm_ can be found even in non-stimulated cells. The increased production of gasdermin D domains was noted when monocytic cells were treated with high concentrations of LPS (10–100 µg/mL). It means that the basic level of GSDMD is stable when cleaved by caspase 1 in response to LPS presence and sufficient for GSDMD_Nterm_-dependent membrane pore formation needed for cytokine release during pyroptosis. In our antibacterial assay, the LPS stimulation was too low for effective bacterial OM permeabilization and enhancement of endolysin activity. This seems to correlate with the role of GSDMD in the immune response to bacterial presence, whereas its overexpression could lead to unfavorable self-cell lysis of monocytic cells.

In conclusion, only recombinant GSDMD_Nterm_ protein was able to efficiently permeabilize *P. aeruginosa* OM and increase endolysin activity against bacteria producing either long or lacking LPS O-chains. The obtained results suggest the limited possibility of using the natural process of pyroptosis occurring in monocytic cells to enhance the bactericidal effect of recombinant phage endolysins against Gram-negative bacteria infection.

## Data Availability

The DNA microarray data have been uploaded to the Jan Kochanowski University repository (https://repozytorium.ujk.edu.pl/dlibra/publication/12832?language=en#description) under Creative Commons Attribution license (CC BY).

## SUPPLEMENTAL MATERIAL

Supplemental material msystems.01106-24-s0001.docx Tables S1-S6; Fig. S1-S4.

ASM does not own the copyrights to Supplemental Material that may be linked to, or accessed through, an article. The authors have granted ASM a non-exclusive, world-wide license to publish the Supplemental Material files. Please contact the corresponding author directly for reuse.
