# Supplementary material for: Exploiting gasdermin-mediated pyroptosis for enhanced antimicrobial activity of phage endolysin against Pseudomonas aeruginosa
Source: mSystems. 2024 Dec 23;10(1):e01106-24. doi: 10.1128/msystems.01106-24 (PMC11748493; doi:10.1128/msystems.01106-24)
Supplement: Supplemental material — Tables S1-S6; Fig. S1-S4. [file msystems.01106-24-s0001.docx]

Exploiting gasdermin-mediated pyroptosis for enhanced antimicrobial activity of phage endolysin against *Pseudomonas aeruginosa*

Dorota Kuc-Ciepluch^1^, Karol Ciepluch^1^, Daria Augustyniak^2^, Grzegorz Guła^2^, Barbara Maciejewska^2^, Artur Kowalik^1, 3^, Ewelina Jop^2^, Zuzanna Drulis-Kawa^2*^, Michał Arabski^1*^

^1^ Division of Medical Biology, Jan Kochanowski University in Kielce, Kielce, Poland

^2^ Department of Pathogen Biology and Immunology, University of Wrocław, Wrocław, Poland

^3^ Department of Molecular Diagnostics, Holy Cross Cancer Centre, Kielce, Poland

Table S1. Top of 15 downregulated genes by *P. aeruginosa* O10 LPS with the highest fold change in the A549 cell line (GO annotations based on the Quick GO).

| FC (fold change) | Gen symbol | GO Term |
| --- | --- | --- |
| 23.82403 | SMAD1-AS1 | heteromeric SMAD protein complex (GO:0071144) |
| 16.941133 | CCL14 | inflammatory response (GO:0006954), extracellular region (GO:0005576), cellular calcium ion homeostasis (GO:0006874), positive regulation of ERK1 and ERK2 cascade (GO:0070374), chemokine-mediated signaling pathway (GO:0070098), cellular response to interferon-gamma (GO:0071346), immune response (GO:0006955), cytokine activity (GO:0005125) chemokine activity (GO:0008009), cellular response to interleukin-1 (GO:0071347), cellular calcium ion homeostasis (GO:0006874) |
| 16.020998 | CCDC89 | cytoplasm (GO:0005737), nucleus (GO:0005634) |
| 14.890545 | MAN2B1 | extracellular region (GO:0005576), lysosome (GO:0005764), mannose metabolic process (GO:0006013), cellular protein modification process (GO:0006464), carbohydrate metabolic process (GO:0005975), metal ion binding (GO:0046872) |
| 13.49492 | KAT2A | nucleus (GO:0005634), chromosome (GO:0005694), cytoplasm (GO:0005737), regulation of transcription, DNA-templated (GO:0006355), histone acetyltransferase activity (GO:0004402), chromatin remodeling (GO:0006338), regulation of T cell activation (GO:0050863), cytokine production (GO:0001816) |
| 11.19738 | CLCA1 | ion transport (GO:0006811), integral component of membrane (GO:0016021),  membrane (GO:0016020), plasma membrane (GO:0005886), intracellular calcium activated chloride channel activity (GO:0005229), proteolysis (GO:0006508) |
| 11.170431 | TRNAU1AP | cytoplasm (GO:0005737), nucleus (GO:0005634), nucleic acid binding (GO:0003676), selenocysteine incorporation (GO:0001514) |
| 10.899675 | ASAH1 | lysosome (GO:0005764), lipid metabolic process (GO:0006629), hydrolase activity (GO:0016787) |
| 10.790707 | ERC2-IT1 | molecular_function (GO:0003674), cellular component (GO:0005575) |
| 9.767274 | ENTPD5 | endoplasmic reticulum (GO:0005783), extracellular region (GO:0005576), hydrolase activity (GO:0016787), protein glycosylation (GO:0006486), protein binding (GO:0005515), regulation of phosphatidylinositol 3-kinase signaling (GO:0014066), ATP metabolic process (GO:0046034) |
| 9.191067 | C1QTNF5 | extracellular region (GO:0005576), membrane (GO:0016021), transport vesicle (GO:0030133), protein secretion (GO:0009306), identical protein binding (GO:0042802), collagen trimer (GO:0005576) |
| 8.033107 | UNC13C | synapse (GO:0045202), plasma membrane (GO:0005886), cytoplasm (GO:0005737), calcium ion binding (GO:0005509), intracellular signal transduction (GO:0035556), metal ion binding (GO:0046872), phospholipid binding (GO:0005543) |
| 7.74313 | PDGFRB | integral component of membrane (GO:0016021), Golgi apparatus (GO:0005794), intracellular membrane-bounded organelle (GO:0043231), G protein-coupled receptor signaling pathway (GO:0007186), cellular response to platelet-derived growth factor stimulus (GO:0036120), protein binding (GO:0005515), positive regulation of calcium ion import (GO:0090280), positive regulation of MAP kinase activity (GO:0043406), ATP binding (GO:0005524) |
| 7.471474 | TIFAB | I-kappaB kinase/NF-kappaB signaling (GO:0007249), inner ear development (GO:0048839), thorax and anterior abdomen determination (GO:0007356), regulation of muscle organ development (GO:0048634) |
| 7.299121 | SLC45A3 | integral component of membrane (GO:0016021), membrane (GO:0016020), transmembrane transport (GO:0055085), positive regulation of glucose metabolic process (GO:0010907), positive regulation of fatty acid biosynthetic process (GO:0045723) |

Table S2. Top of 15 upregulated genes by *P. aeruginosa* O10 LPS with the highest fold change in the HeLa cell line (GO annotations based on the Quick GO).

| FC (fold change) | Gen symbol | GO Term |
| --- | --- | --- |
| 4.714636 | WFDC9 | extracellular space (GO:0005615), innate immune response (GO:0045087), antibacterial humoral response (GO:0019731), negative regulation of endopeptidase activity (GO:0010951), serine-type endopeptidase inhibitor activity (GO:0004867), protein binding (GO:0005515) |
| 3.765462 | OR51D1 | integral component of membrane (GO:0016021), membrane (GO:0016020), signal transduction (GO:0007165), plasma membrane (GO:005886), response to stimulus (GO:0050896), G protein-coupled receptor signaling pathway (GO:0007186), G protein-coupled receptor activity (GO:0004930) |
| 3.6745274 | GPR78 | integral component of membrane (GO:0016021), membrane (GO:0016020), G protein-coupled receptor signaling pathway (GO:0007186), G protein-coupled receptor activity (GO:0004930), adenylate cyclase-activating G protein-coupled receptor signaling pathway (GO:0007189), protein binding (GO:0005515), signal transduction (GO:0007165) |
| 3.4771352 | TRIM77 | cytoplasm (GO:0005737), metal ion binding (GO:0046872), zinc ion binding (GO:0008270), protein ubiquitination (GO:0016567), ubiquitin protein ligase activity (GO:00616030) |
| 3.349552 | HTR3E | integral component of membrane (GO:0016021), membrane (GO:0016020), ion transport (GO:0006811), ion transmembrane transport (GO:0034220), ion channel activity (GO:0005216), transmembrane signaling receptor activity (GO:0004888), extracellular ligand-gated ion channel activity (GO:0005230), serotonin receptor signaling pathway (GO:0007210), regulation of membrane potential (G O:0042391), protein binding (GO:0005515) |
| 3.3216622 | IMPG2 | integral component of membrane (GO:0016021), membrane (GO:0016020), extracellular matrix structural constituent (GO:0005201), hyaluronic acid binding (GO:0005540), heparin binding (GO:0008201), receptor complex (GO:0043235) |
| 3.2906222 | TMPRSS7 | integral component of membrane (GO:0016021), membrane (GO:0016020), plasma membrane (GO:005886), peptidase activity (GO:0008233), proteolysis (GO:0006508), serine-type endopeptidase, activity (GO:0004252), hydrolase activity (GO:0016787) |
| 3.2688327 | ANGPT4 | extracellular space (GO:005615), signal transduction (GO:0007165), negative regulation of apoptotic process (GO:0043066), positive regulation of peptidyl-tyrosine phosphorylation (GO:0050731), transmembrane receptor protein tyrosine kinase activator activity (GO:0030297), receptor tyrosine kinase binding (GO:0030971), activation of transmembrane receptor protein tyrosine kinase activity (GO:0007171), angiogenesis (GO:0001525), signaling receptor binding (GO:0005102) |
| 3.2301133 | FERD3L | nucleus (GO:0005634), DNA binding (GO:0003677), regulation of transcription by RNA polymerase II (GO:0006357), RNA polymerase II transcription regulatory region sequence-specific DNA binding (GO:0000977), DNA-binding transcription repressor activity, RNA polymerase II-specific (GO:0001227), protein dimerization activity (GO:0046983), protein binding (GO:0005515) |
| 3.22568 | OR8U1 | integral component of membrane (GO:0016021), membrane (GO:0016020), plasma membrane (GO:005886), signal transduction (GO:0007165), response to stimulus (GO:0050896), G protein-coupled receptor signaling pathway (GO:0007186), G protein-coupled receptor activity (GO:0004930) |
| 3.2095532 | PROKR2 | integral component of membrane (GO:0016021), membrane (GO:0016020), plasma membrane (GO:005886), signal transduction (GO:0007165), G protein-coupled receptor signaling pathway (GO:0007186), G protein-coupled receptor activity (GO:0004930), peptide binding (GO:0042277) can promote angiogenesis and induce strong gastrointestinal smooth muscle contraction |
| 3.2019274 | GALR2 | integral component of membrane (GO:0016021), membrane (GO:0016020), signal transduction (GO:0007165), G protein-coupled receptor signaling pathway (GO:0007186), G protein-coupled receptor activity (GO:0004930), G protein-coupled peptide receptor activity (GO:0008528), positive regulation of transcription by RNA polymerase II (GO:0045944), protein binding (GO:0005515), cell surface receptor signaling pathway (GO:0007166)neuromodulator present in the brain, gastrointestinal system, and hypothalamopituitary axis |
| 3.1823707 | SOX10 | nucleus (GO:0005634), DNA binding (GO:00036777), DNA-binding transcription factor activity, RNA polymerase II-specific (GO:0000981), positive regulation of transcription by RNA polymerase II (GO:0045944), negative regulation of apoptotic process (GO:0043066), membrane (GO:0016020), cytoplasm (GO:0005737) |
| 3.128601 | GRK5-IT1 | G protein-coupled receptor kinase activity (GO:0004703) |
| 3.12537 | GAPT | integral component of membrane (GO:0016021), membrane (GO:0016020), plasma membrane (GO:005886), immunoglobulin production involved in immunoglobulin mediated immune response (GO:0002381), B cell proliferation involved in immune response (GO:0002322), B cell homeostasis (GO:0001782) |

Table S3. Top of 15 downregulated genes by *P. aeruginosa* O10 LPS with the highest fold change in the HeLa cell line (GO annotations based on the Quick GO).

| FC (fold change) | Gen symbol | GO Term |
| --- | --- | --- |
| 18,550001 | SSBP3-AS1 | heteromeric SMAD protein complex (GO:0071144) |
| 8.084809 | LIN28A | nucleic acid binding (GO:0003676), zinc ion binding (GO:0008270), positive regulation of protein kinase B signaling (GO:0051897), regulation of gene silencing by miRNA (GO:0060964), nucleus (GO:0005634), cytoplasm (GO:0005737), polysome (GO:0005834), pre-miRNA processing (GO:0031054), positive regulation of TOR signaling (GO:0032008) |
| 6.446257 | CLCA1 | ion transport (GO:0006811), integral component of membrane (GO:0016021), membrane (GO:0016020), plasma membrane (GO:0005886), intracellular calcium activated chloride channel activity (GO:0005229), proteolysis (GO:0006508) |
| 6.1400256 | C1QTNF5 | extracellular region (GO:0005576), apical plasma membrane (GO:0016324), membrane (GO:0016020), transport vesicle (GO:0030133), protein secretion (GO:0009306), identical protein binding (GO:0042802) |
| 5.7542586 | UNC13C | synapse (GO:0045202), chemical synaptic transmission (GO:0007268), diacylglycerol binding (GO:0019992) |
| 5.6120934 | EFCC1 | cytosol (GO:0005829), calcium ion binding (GO:0005509) |
| 5.580892 | KCNAB3 | cytoplasm (GO:0005737), integral component of membrane (GO:0016021), regulation of ion transmembrane transport (GO:0034765), potassium ion transport (GO:0006813), voltage-gated potassium channel activity (GO:0005242) |
| 5.348129 | BCL6B | nucleus (GO:0005634) DNA binding (GO:0003677), negative regulation of transcription by RNA polymerase II (GO:0000122), RNA polymerase II transcription regulatory region sequence-specific DNA binding (GO:0000977), DNA-binding transcription repressor activity, RNA polymerase II-specific (GO:0001227), type 2 immune response (GO:0042092), regulation of inflammatory response (GO:0050727), regulation of cytokine production (GO:0001817) |
| 5.3276715 | SHC4 | intracellular signal transduction (GO:0035556), receptor tyrosine kinase binding (GO:0030971), positive regulation of cell population proliferation (GO:0008284), apoptotic process (GO:0006915), regulation of gene expression (GO:0010468), stem cell differentiation (GO:0048863), plasma membrane (GO:0005886), protein kinase binding (GO:0019901) |
| 5.1697407 | LIM2 | integral component of membrane (GO:0016021), membrane (GO:0016020), structural constituent of eye lens (GO:0005212) |
| 5.1686816 | NYAP1 | phosphatidylinositol 3-kinase signaling (GO:0014065), neuron projection morphogenesis (GO:0048812) |
| 4.825547 | UNC5B | integral component of membrane (GO:0016021), membrane (GO:0016020), plasma membrane (GO:0005886), angiogenesis (GO:0001525), apoptotic process (GO:0006915), negative regulation of extrinsic apoptotic signaling pathway in absence of ligand (GO:2001240), protein binding (GO:0005515), signal transduction (GO:0007165) |
| 4.762355 | ABCA10 | integral component of membrane (GO:0016021), membrane (GO:0016020), transmembrane transport (GO:0055085), ATPase-coupled transmembrane transporter activity (GO:0042626) |
| 4.5368357 | APOL3 | cytoplasm (GO:0005737), inflammatory response (GO:0006954), lipid transport (GO:0006869), lipid binding (GO:0008289), positive regulation of I-kappaB kinase/NF-kappaB signaling (GO:0043123) |
| 4.4895086 | TRPV4 | integral component of plasma membrane (GO:0005887), integral component of membrane (GO:0016021), ion transport (GO:0006811), osmosensory signaling pathway (GO:0007231), ATP binding (GO:0005524), protein binding (GO:0005515), lipid binding (GO:0008289) |

Table S4. Top of 15 upregulated genes by *P. aeruginosa* O10 LPS with the highest fold change in THP1-Xblue cell line (GO annotations based on the Quick GO).

| FC (fold change) | Gen symbol | GO Term |
| --- | --- | --- |
| 227.11972 | CXCL13 | cellular response to lipopolysaccharide (GO:0071222), inflammatory response (GO:0006954), immune response (GO:0006955), integral component of membrane (GO:0016021), membrane (GO:0016020), chemokine-mediated signaling pathway (GO:0070098), antimicrobial humoral immune response mediated by antimicrobial peptide (GO:0061844), cytokine activity (GO:0005125) |
| 87.13244 | CXCL3 | cellular response to lipopolysaccharide (GO: 007122), inflammatory response (GO:0006954), immune response (GO:0006955), CXCR chemokine receptor binding (GO:0045236), chemokine activity (GO:0008009), antimicrobial humoral immune response mediated by antimicrobial peptide (GO:0061844), cytokine activity (GO:0005125), killing of cells of other organism (GO:0031640) |
| 76.35865 | CCL2 | chemokine (C-C motif) ligand 2 secretion (GO:0035906), positive regulation of chemokine (C-C motif) ligand 2 secretion (GO:1904209) |
| 72.20718 | Il1B | response to lipopolysaccharide (GO:0032496), immune response (GO:0006955), inflammatory response (GO:0006954), cytokine activity (GO:0005125), interleukin-1 receptor region (GO:0005149), extracellular region (GO:0005576), extracellular space (GO:0005615), signal transduction (GO:0007165), positive regulation of interleukin-6 production (GO:0032755), positive regulation of p38MAPK cascade (GO:1900745), positive regulation of I-kappaB kinase/NF-kappaB signaling (GO:0043123), positive regulation of interleukin-8 production (GO:0032757) |
| 69.505295 | RGS1 | response to bacterium (GO:0009617), plasma membrane (GO:0005886), G protein-coupled receptor signaling pathway (GO:0007186), GTPase activator activity (GO:0005096), signal transduction (GO:0007165), positive regulation of GTPase activity (GO:0043547) |
| 58.763435 | MIR146A | gene silencing by miRNA (GO:0035195) |
| 53.12312 | TNFAIP6 | negative regulation of inflammatory response (GO:0050728), hyaluronic acid binding (GO:00055400), cell adhesion (GO:0007155), inflammatory response (GO:0006954), cell-cell signaing (GO:0007267), protein binding (GO:0005515), extracellular region (GO:0005576), extracellular space (GO:0005615) |
| 51.89944 | CCL8 | immune response (GO:0006955), inflammatory response (GO:0006954), antimicrobial humoral immune response mediated by antimicrobial peptide (GO:0061844), cytokine activity (GO:0005125), chemokine activity (GO:0008009), cellular response to interferon-gamma (GO:0071346), signal transduction (GO:0007165), cellular response to interleukin-1 (GO:0071347), positive regulation of ERK1 and ERK2 cascade (GO:0070374), killing of cells of other organism (GO:0031640) |
| 49.94572 | CXCL8 | cellular response to lipopolysaccharide (GO:0071222), immune response (GO:0006955), inflammatory response (GO:0006954), interleukin-8 receptor binding (GO:0005153), cytokine activity (GO:0005125), chemokine activity (GO:0008009), neutrophil activation (GO:0042119), defense response (GO:0006952), regulation of entry of bacterium into host cell (GO:2000535), negative regulation of G protein-coupled receptor signaling pathway (GO:0045744), antimicrobial humoral immune response mediated by antimicrobial peptide (GO:0061844) |
| 46.76053 | MPP9 | Golgi membrane (GO:0000139), Golgi apparatus (GO:0005794), centriole (GO:0005814), cytoskeleton (GO:0005856), cytoplasm (GO:0005737), membrane (GO0016020) |
| 43.24729 | CCL7 | immune response (GO:0006955), cytokine activity (GO:0005125), chemokine activity (GO:0008009), signal transduction (GO:0007165), extracellular region (GO:0005576) |
| 40.52647 | IL6 | defense response to Gram-negative bacterium (GO:0050829), cellular response to lipopolysaccharide (GO:0071222), immune response (GO:0006955), inflammatory response (GO:0006954), positive regulation of apoptotic process (GO:0043065), positive regulation of MAPK cascade (GO:0043410), positive regulation of receptor signaling pathway *via* STAT (GO:1904894), receptor signaling pathway *via* JAK-STAT (GO:0007259), positive regulation of apoptotic process (GO:0043065), positive regulation of immunoglobulin secretion (GO:0051024), positive regulation of B cell activation (GO:0050871), positive regulation of T cell proliferation (GO:0042102), positive regulation of NF-kappaB transcription factor activity (GO:0051092) |
| 35.368835 | CXCL1 | immune response (GO:0006955), cellular response to lipopolysaccharide (GO:0071222), inflammatory response (GO:0006954), cytokine activity (GO:0005125) chemokine activity (GO:0008009), G protein-coupled receptor signaling pathway (GO:0007186), antimicrobial humoral immune response mediated by antimicrobial peptide (GO:0061844), killing of cells of other organism (GO:0031640) |
| 29.506351 | SOCS3 | negative regulation of inflammatory response (GO:0050728), interleukin-6-mediated signaling pathway (GO:0070102), negative regulation of apoptotic process (GO:0043166), receptor signaling pathway *via* JAK-STAT (GO:0007259), negative regulation of tyrosine phosphorylation of STAT protein (GO:0042532), intracellular signal transduction (GO:0035556) |
| 27.159191 | CCL20 | cellular response to interleukin-1 (GO:0071347), inflammatory response (GO:0006954), immune response (GO:0006955), antimicrobial humoral immune response mediated by antimicrobial peptide (GO:0061844), killing of cells of other organism (GO:0031640), cytokine activity (GO:0005125), chemokine activity (GO:0008009), CCR chemokine receptor binding (GO:0048020), calcium-mediated signaling using intracellular calcium source (GO:0035584) |

Table S5. Top of 15 downregulated genes by *P. aeruginosa* O10 LPS with the highest fold change in THP1-Xblue cell line (GO annotations based on the Quick GO).

| FC (fold change) | Gen symbol | GO Term |
| --- | --- | --- |
| 24.32195 | MS4A3 | integral component of membrane (GO:0016021), membrane (GO:0016020), regulation of cell cycle (GO:0051726), cytoplasm (GO:0005737) |
| 13.624988 | SERPINB10 | extracellular space (GO:005615), nucleoplasm (GO:005654), cytosol (GO:0005859), negative regulation of apoptotic process (GO:0043066), negative regulation of endopeptidase activity (GO:0010951), nucleus (GO:0005634) |
| 12.340872 | GPA33 | integral component of membrane (GO:0016021), membrane (GO:0016020), integral component of plasma membrane (GO:0005877), signaling receptor activity (GO:0038023), protein binding (GO:0005515) |
| 12.235304 | CLEC12A | integral component of membrane (GO:0016021), membrane (GO:0016020), plasma membrane (GO:0005886), carbohydrate binding (GO:0030246), neutrophil degranulation (GO:0043312) |
| 11.841952 | CNR1 | integral component of membrane (GO:0016021), plasma membrane (GO:005886), G protein-coupled receptor signaling pathway (GO:0007186), G protein-coupled receptor activity (GO:0004930), cannabinoid signaling pathway (GO:0038171), signal transduction (GO:0007165), protein binding (GO:0005515), positive regulation of acute inflammatory response to antigenic stimulus (GO:0002866), negative regulation of ion transport (GO:0043271), positive regulation of apoptotic process (GO:0043065), response to lipopolysaccharide (GO:0032496) |
| 8.6170025 | DEPDC4 | intracellular signal transduction (GO:0035556) |
| 7.5374727 | SLC40A1 | integral component of membrane (GO:0016021), plasma membrane (GO:005886), membrane (GO:0016020), nucleoplasm (GO:005654), cytosol (GO:0005859), ion transport (GO:0006811), protein binding (GO:0005515), negative regulation of apoptotic process (GO:0043066), lymphocyte homeostasis (GO:0002260) |
| 7.0772705 | TMOD1 | cytoskeleton (GO:0005856), cytosol (GO:0005859), cytoplasm (GO:0005737), membrane (GO:0016020), COP9 signalosome (GO: 0008180), pointed-end actin filament capping (GO:0051649), actin filament binding (GO:0051015), tropomyosin binding (GO:0005523), cortical cytoskeleton (GO:0030863) |
| 6.6002007 | DSC3 | integral component of membrane (GO:0016021), membrane (GO:0016020), plasma membrane (GO:005886), homophilic cell adhesion *via* plasma membrane adhesion molecules (GO:0007156), calcium ion binding (GO:005509), cell adhesion (GO:007155) |
| 6.5236998 | NMUR1 | integral component of membrane (GO:0016021), membrane (GO:0016020), plasma membrane (GO:005886), G protein-coupled receptor signaling pathway (GO:0007186), calcium-mediated signaling (GO:0019722), inositol phosphate-mediated signaling (GO:0048016), chloride transport (GO:0006821), neuropeptide signaling pathway (GO:007218), signal transduction (GO:0007165) |
| 6.357195 | RET | integral component of membrane (GO:0016021), membrane (GO:0016020), protein kinase activity (GO:0004672), ATP binding (GO:0005524), protein phosphorylation (GO:0006468), MAPK cascade (GO:0000165), calcium ion binding (GO:0005509) |
| 5.834671 | CLRN1 | integral component of membrane (GO:0016021), membrane (GO:0016020), trans-Golgi network transport vesicle (GO:0030140), cell motility (GO:0048870) |
| 5.7966366 | F13A1 | protein-glutamine gamma-glutamyltransferase activity (GO:0003810), peptide cross-linking (GO:0018149), metal ion binding (GO:0046872), cytoplasm (GO:0005737), extracellular region (GO:0005576) |
| 5.7462707 | SERPINI2 | extracellular space (GO:0005615), integral component of membrane (GO:0016021), membrane (GO:0016020), signal transduction (GO:0007165), serine-type endopeptidase inhibitor activity (GO:0004867), regulation of cell adhesion (GO:0030155), protein binding (GO:0005515) |
| 5.475106 | MS4A6A | integral component of membrane (GO:0016021), membrane (GO:0016020) |

Table S6. Activation of the classical complement pathway (CH50) in human serum preincubated with LPS

| LPS | PAO1 | OO38  (non-CF) | CF217 | CF532 | CF832 | no LPS  (control -) | O10  (control +) |
| --- | --- | --- | --- | --- | --- | --- | --- |
| CH50 titer (AU)  Mean SD | 47 4 | 68 2 | 90 11 | 67 4 | 70 3 | 87 9 | 29 6 |

The results represent at least 3 independent biological repeats each with two technical replicates. The properties of LPS to sequestrate complement components were studied by measuring the 50% haemolytic complement (CH50) activity of human serum. This step aimed to evaluate the "residual" hemolytic activity (RHA) of complement in human serum pre-incubated with tested LPS. The hypothesis tested here was that LPS binding and activating complement more strongly would lead to greater complement consumption, resulting in weaker "residual" activity in the hemolytic test. Alternatively, weaker complement-binding and activating LPS would retain a strong potential for hemolysis of IgG-opsonized SRBCs. As shown in Table S6, the weakest consumer of complement was LPS from the CF217 strain (rough form). The residual CH50 titer that remained in the serum after its prior activation by LPS of this strain was as high as 90 AU, and was on the level of non-treated active serum (negative control).

Table S7. Bactericidal activity of normal sheep serum against clinical *P. aeruginosa* strains.

| 50% NSS | Strain | T0 | T1h | T2h |
| --- | --- | --- | --- | --- |
|  | PAO1 | 5.3 × 10^5^ 4.7 × 10^4^ | 7.3 × 10^5^ 1.9 × 10^5^ | 1.7 × 10^6^ 3.9 × 10^4^ |
|  | non-CF 0038 | 4.5 × 10^5^ 1.1 × 10^5^ | 5.1 × 10^5^ 5.9 × 10^4^ | 1.2 × 10^6^ 2.6 × 10^4^ |
|  | CF217 | 7.2 × 10^5^ 2.8 × 10^5^ | <10^2^ | <10^2^ |
|  | CF532 | 2.3 × 10^5^ 6.4 × 10^4^ | <10^2^ | <10^2^ |
|  | CF832 | 7.4 × 10^5^ 2.4 × 10^5^ | 2.2 × 10^5^ 3.6 × 10^4^ | 5.6 × 10^4^ 1.6 × 10^4^ |
| 50% HiNSS* | PAO1 | 6.3 × 10^5^ 3.8 × 10^4^ | 9.3 × 10^5^ 3.5 × 10^4^ | 2.4 × 10^6^ 1.8 × 10^5^ |
|  | non-CF 0038 | 5.4 × 10^5^ 3.4 × 10^5^ | 9.7 × 10^5^ 9.4 × 10^4^ | 2.3 × 10^6^ 3.1 × 10^5^ |
|  | CF217 | 1.3 × 10^6^ 3.6 × 10^5^ | 3.3 × 10^6^ 2.2 × 10^6^ | 4.6 × 10^6^ 2.0 × 10^6^ |
|  | CF532 | 3.0 × 10^5^ 7.8 × 10^4^ | 4.8 × 10^5^ 5.1 × 10^4^ | 7.0 × 10^5^  2.9 × 10^5^ |
|  | CF832 | 1.2 × 10^6^ 8.4 × 10^5^ | 1.5 × 10^6^ 7.7 × 10^5^ | 2.6 × 10^6^ 1.6 × 10^6^ |

Results are given as cfu/ml SD. NSS – active normal sheep serum; * HiNSS – heat-inactivated sheep serum (56°C, 60 min). The results represent 2 independent biological repeats.


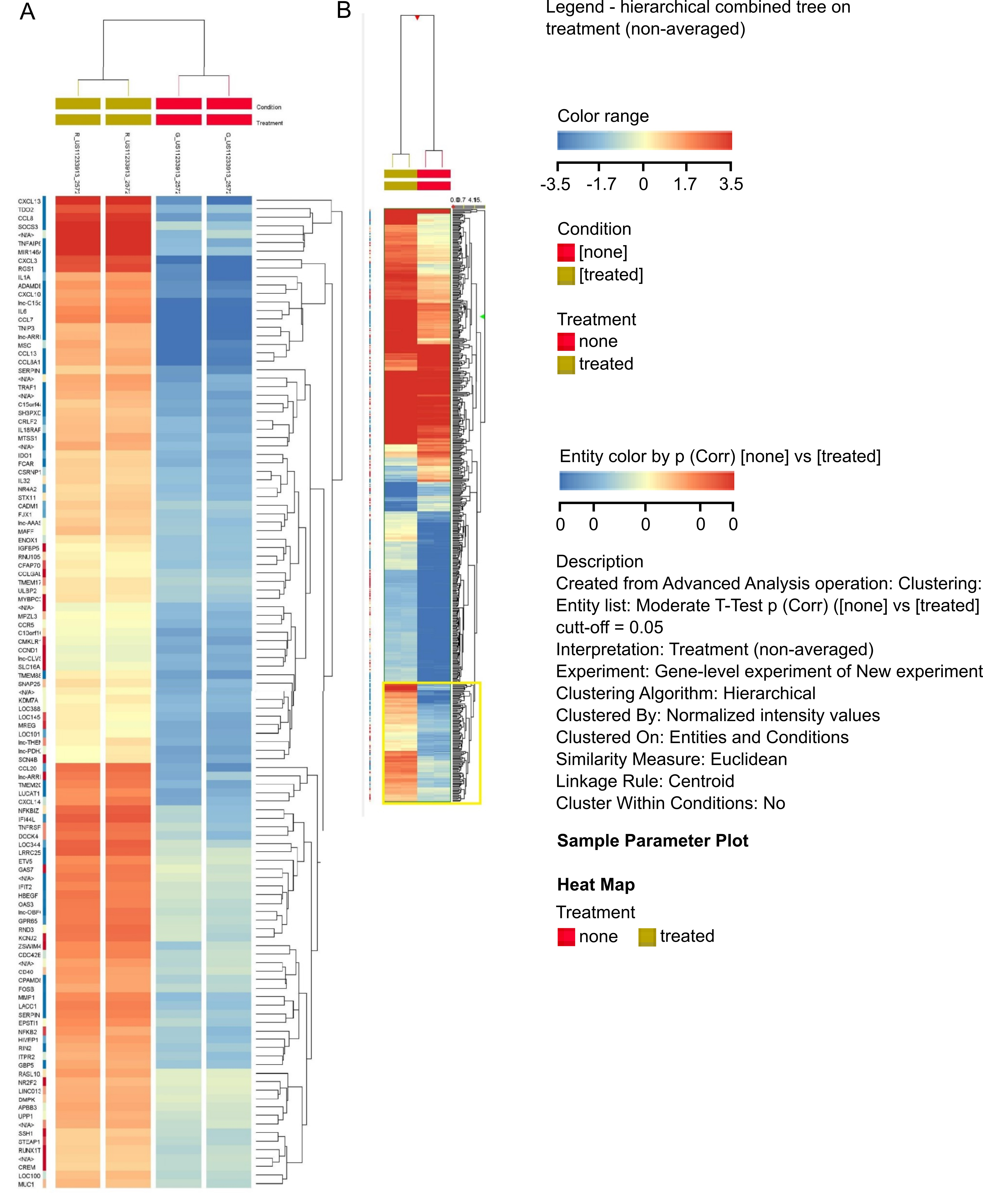


Fig. S1. Heatmaps of the gene expression profile of THP1-Xblue cells induced by 0.1 µg/ml *P. aeruginosa* O10 LPS, generated from DNA microarray data using GeneSpring GX software. The condition treatments indicate the untreated sample (red boxes), and the sample treated with *P. aeruginosa* LPS (green boxes). The red color in the heatmaps means an increase in gene expression level, whereas the blue color shows a decrease in gene expression of the treated sample relative to the control sample. The intensity of both colors depends on the level of the fold change. Panel A represents the whole gene expression change profile. The yellow squares at the top show the gene expression with the highest fold change. Panel B is the zoom-in of the gene expression with the highest fold change analyzed in two repeats. The details are included in Table S4, Supporting data.


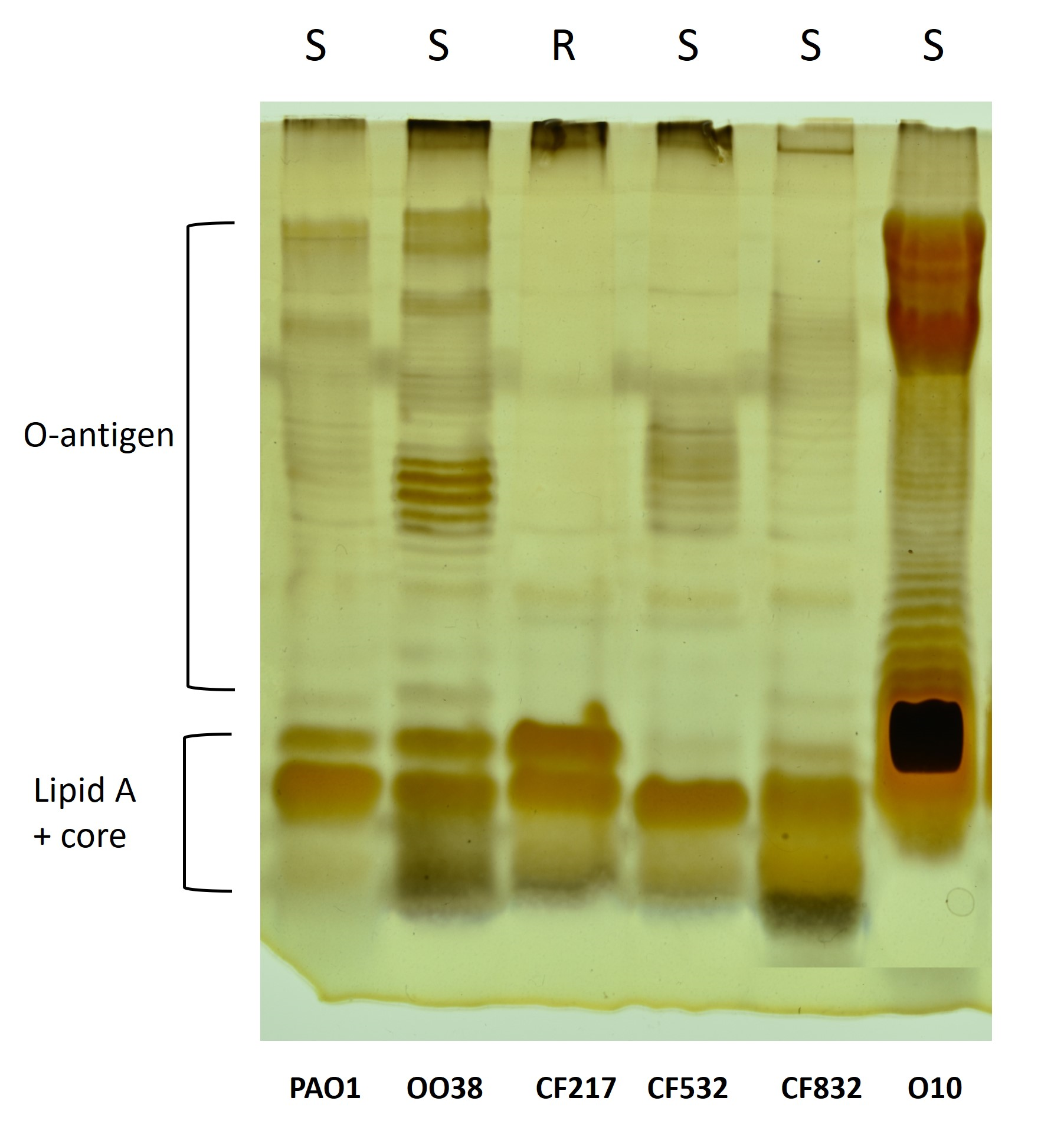


Fig. S2. The LPS patterns of *P. aeruginosa* PA O10, PAO1, CF532, CF832, CF217, and non-CF0038 strains. S – *smooth* form of LPS; R – *rough* form of LPS


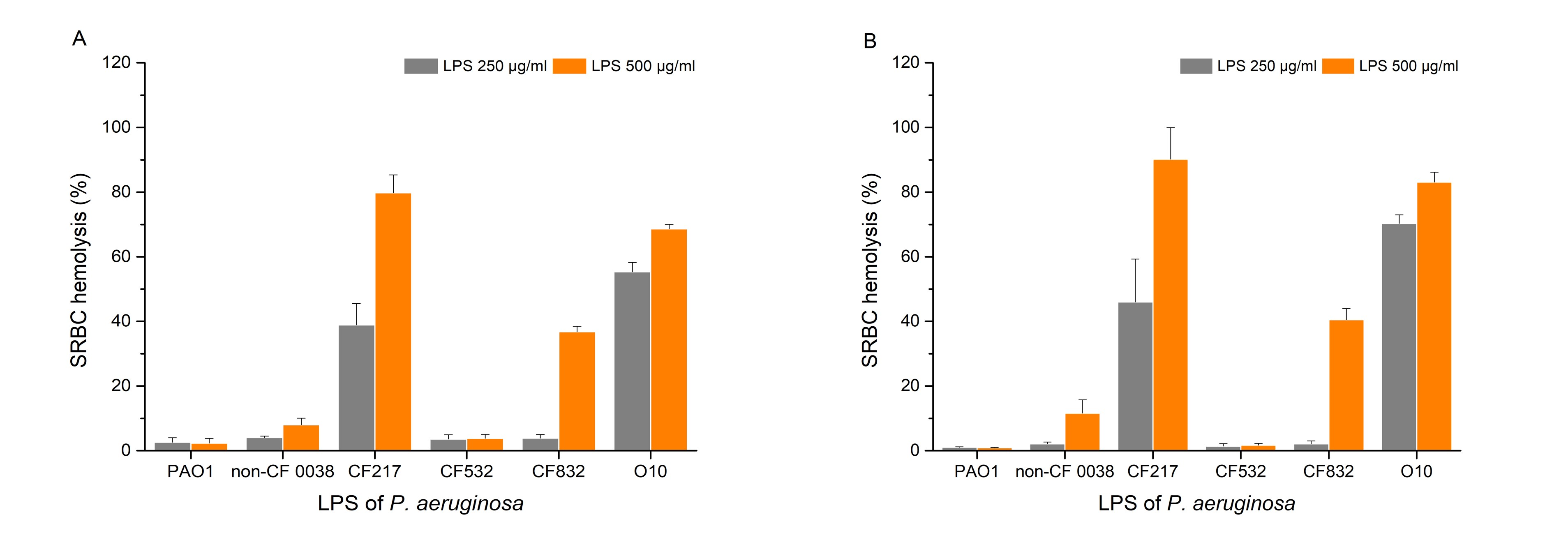


Fig. S3. Hemolytic potential of *P. aeruginosa* LPS against sheep red blood cells (SRBC). The standard curve, which is the relationship between absorbance and percentage of hemolysis, was used to calculate the degree of SRBC hemolysis induced by LPS. Data showing hemolysis after 15 min of treatment (A) or 2 h of treatment (B) are expressed as mean ± SEM from two independent experiments performed in duplicates.


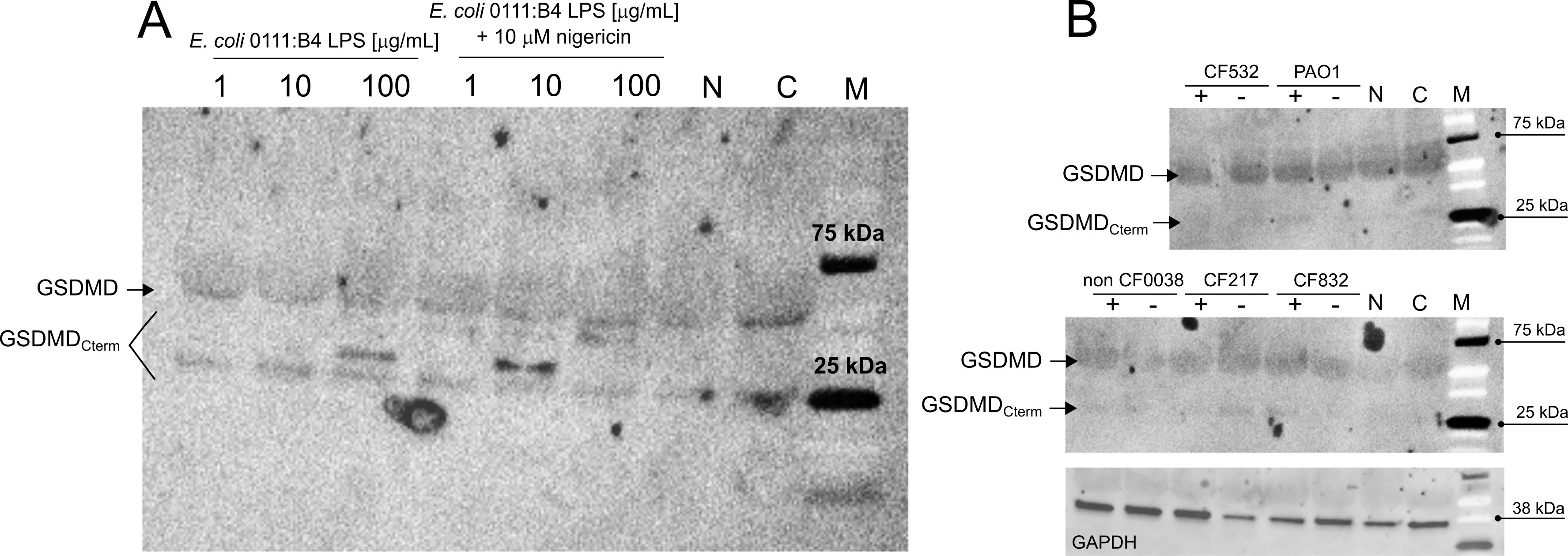


Fig. S4. (A) Western Blot analysis for GSDMD (53 kDa) protein and its cleavage product GSDMD_Cterm_ (22 kDa) under reducing conditions with β-mercaptoethanol in THP1-Null2 cells in supernatants after treatment with *E. coli* 0111:B4 LPS at three different concentrations 1 µg/ml, 10 µg/ml, 100 µg/ml with (+) or without (-) nigericin (10 µM); (B) Western Blot analysis for GSDMD protein and its cleavage product GSDMD_Cterm_ under reducing conditions with β-mercaptoethanol in THP1-Null2 cells in supernatants after treatment with LPS of different *P. aeruginosa* at a concentration of 1 µg/ml and with (+) or without (-) nigericin (10 µM). The GAPDH was used as a control protein to normalize the target protein. C- control, M-mass marker; N – nigericin
